# Salicylic diamines selectively eliminate residual undifferentiated cells from pluripotent stem cell-derived cardiomyocyte preparations

**DOI:** 10.1038/s41598-021-81351-z

**Published:** 2021-01-27

**Authors:** Karsten Burkert, Hadiseh Taheri, Sarkawt Hamad, Matteo Oliverio, Gabriel Peinkofer, Jan-Wilhelm Kornfeld, Wacharee Harnying, Kurt Pfannkuche, Jürgen Hescheler, Albrecht Berkessel, Tomo Šarić

**Affiliations:** 1 Center for Physiology and Pathophysiology, Institute for Neurophysiology, Medical Faculty, University of Cologne, Cologne, Germany; 2 Max Planck Institute for Metabolism Research, Cologne, Germany; 3 Department of Internal Medicine III, University Hospital Cologne, Cologne, Germany; 4 Department of Chemistry, Organic Chemistry, University of Cologne, Cologne, Germany

**Keywords:** Small molecules, Induced pluripotent stem cells

## Abstract

Clinical translation of pluripotent stem cell (PSC) derivatives is hindered by the tumorigenic risk from residual undifferentiated cells. Here, we identified salicylic diamines as potent agents exhibiting toxicity to murine and human PSCs but not to cardiomyocytes (CMs) derived from them. Half maximal inhibitory concentrations (IC_50_) of small molecules SM2 and SM6 were, respectively, 9- and 18-fold higher for human than murine PSCs, while the IC_50_ of SM8 was comparable for both PSC groups. Treatment of murine embryoid bodies in suspension differentiation cultures with the most effective small molecule SM6 significantly reduced PSC and non-PSC contamination and enriched CM populations that would otherwise be eliminated in genetic selection approaches. All tested salicylic diamines exerted their toxicity by inhibiting the oxygen consumption rate (OCR) in PSCs. No or only minimal and reversible effects on OCR, sarcomeric integrity, DNA stability, apoptosis rate, ROS levels or beating frequency were observed in PSC-CMs, although effects on human PSC-CMs seemed to be more deleterious at higher SM-concentrations. Teratoma formation from SM6-treated murine PSC-CMs was abolished or delayed compared to untreated cells. We conclude that salicylic diamines represent promising compounds for PSC removal and enrichment of CMs without the need for other selection strategies.

## Introduction

Myocardial infarction (MI) is the leading cause of death and disability worldwide^1^. Transplantation of pluripotent stem cell-derived cardiomyocytes (PSC-CMs) into the infarcted heart holds great promise as an alternative therapeutic strategy for these patients^2–4^. However, a major obstacle for clinical use of PSC-CMs is the tumor formation from residual undifferentiated cells^5^. Even a few contaminating PSCs could lead to teratoma formation and represent a serious safety risk to patients^6^. Thus, it is crucial to completely eliminate undifferentiated PSCs from therapeutic cell preparations to ensure their safe application in the clinic.

Numerous groups developed transgenic PSC lines expressing selection markers under lineage-specific promoters to enable removal of PSCs and purify desired tissue-specific cell populations^7,8^. However, genetic modification required for this approach may itself lead to mutagenesis and increase the hurdle for clinical use of such cells. To overcome this problem, elimination strategies based on the unique molecular properties of PSCs have been developed. They include immunological^9–14^, biophysical^15–18^, biochemical^19–22^, metabolic^23–26^ and genetic approaches^18,27–34^. Among them, small synthetic molecules (SMs) represent powerful tools because they offer simplicity, accessibility, scalability and flexibility in combinatorial application at specific times, durations and doses, which represents an advantage compared to genetic and biologic approaches. Although several SMs have already demonstrated their utility for selective PSC removal^35–42^, rigorous assessment of their effects on differentiated cells has not been performed in most of these studies and not all available SMs might be equally suitable for purging of PSCs from different PSC-derivatives.

Here, we identify salicylic diamines as novel SMs capable of killing PSCs in vitro at half maximal inhibitory concentrations (IC_50_) in the range of 0.2–1.1 μM for murine PSC (mPSCs) and 0.3–12.5 μM for human induced PSCs (hiPSCs). All tested SMs exerted this activity by inhibiting mitochondrial function which was more pronounced in murine than in human PSCs most likely reflecting their different reliance on oxidative phosphorylation (OXPHOS) for energy production. These compounds exhibited no or minimal side-effects on PSC-CMs at concentrations that were toxic to undifferentiated cells. Most importantly, the compound SM6 eliminated undifferentiated PSCs and enriched CMs in mPSC differentiation cultures to a similar extent as puromycin in a genetic selection approach. In addition, SM6 alone produced more heterogeneous mPSC-CM populations by preserving CM subtypes that were otherwise eliminated by puromycin. Thus, salicylic diamines such as SM6 and its derivatives represent efficient agents for PSC-elimination in CM preparations, thereby decreasing the risk of their teratogenicity upon transplantation in preclinical animal studies and future clinical trials.

## Results

### Salicylic diamines are cytotoxic to murine and human PSCs

**Figure 1:**
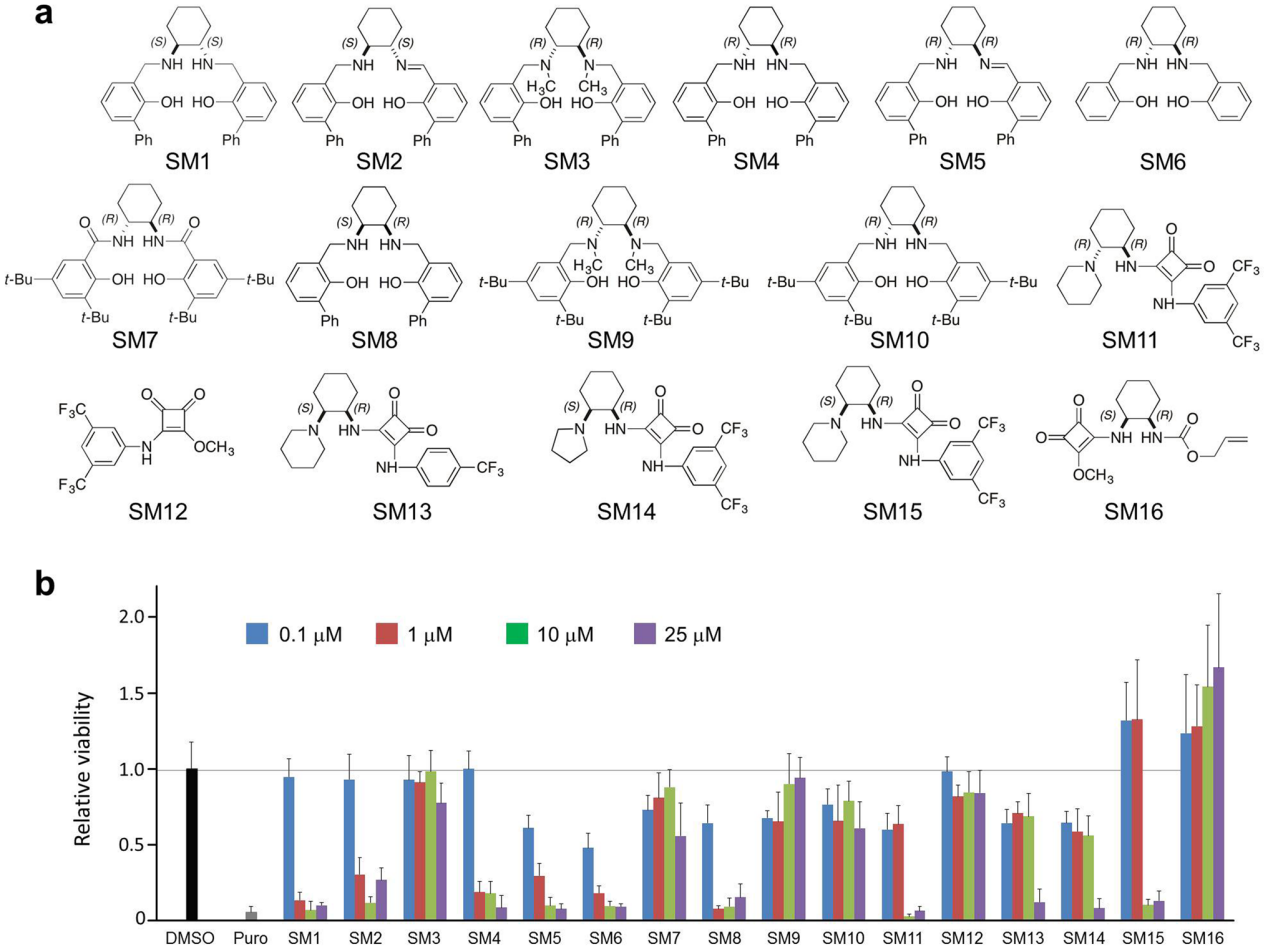
Toxicity of small molecules SM1-SM16 against miPSC line aPIG-AT25. (**a**) Chemical structures of 10 salicylic diamines (SM1-10) and 6 square amides (SM11-16) used in a cytotoxicity screen. (**b**) Relative viability of miPSCs after 48 h of treatment with the indicated concentrations of SMs (mean ± SD; n = 4). Control cells were treated with 0.125% DMSO or 8 μg/ml puromycin (Puro). Similar results were obtained in two additional independent experiments.

In an attempt to reduce the teratogenicity of PSC-CM preparations in animal experiments, we found that the previously described inhibitor PluriSIn #1^36^ has a low cytotoxic activity against mPSCs in vitro (Supplementary Fig. S1, Supplementary Results). Searching for more potent PSC-eliminating SMs, we analyzed the collection of ten salicylic diamines, termed SM1-SM10, and six squaric amides, termed SM11-SM16, for their cytotoxicity against PSCs (Fig. 1a). The choice for these SMs was based on our previous observation that salicylic diamines can selectively induce apoptosis in leukemia and lymphoma cells and even overcome multidrug resistance^43,44^. Since PSCs share some characteristics with cancer stem cells^45–47^, we reasoned that these compounds might also exert selective toxicity against PSCs but not their differentiated derivatives.

Cytotoxicity screens with transgenic miPSC line αPIG-AT25^48^ revealed that SM1, SM2, SM4, SM5, SM6 and SM8 were the most effective and killed these cells in a concentration-dependent manner. After 48 h of treatment with 1 µM of these SMs more than 70% of PSCs were eliminated while an almost complete cell killing was achieved at 10 and 25 µM concentrations (Fig. 1b). Dose–response curves obtained with three different hiPSC lines (Supplementary Tables S1 and S2) and three different mPSC lines (Table S3) revealed that the most effective salicylic diamines displayed cytotoxic activity against mPSCs with a half maximal inhibitory concentration (IC_50_) ranging from 0.20 ± 0.17 µM for SM2 and 0.24 ± 0.18 µM for SM6 to 1.1 ± 0.4 µM for SM8 while the IC_50_ for hiPSCs was in similar range for SM8 (~ 0.4 µM) but much higher for SM2 and SM6 ranging, respectively, from 2.7–11.0 µM and 1.6–12.5 µM for different hiPSC lines (Supplementary Tables S2 and S3, and Fig. S2). Further analyses performed with new batches of SM2, SM6 and SM8 corroborated initial findings and validated their toxicity towards miPSCs (Supplementary Fig. S3a). The analysis of the structure–function relationship for one of the most potent compounds SM6 revealed that its cytotoxic activity is independent of the spatial orientation of its atoms and that the intact SM6 molecule is required for its PSC-toxicity (Supplementary Fig. S3b and S3c, Supplementary Results).

### Salicylic diamines do not affect the viability of iPSC-CMs

**Figure 2:**
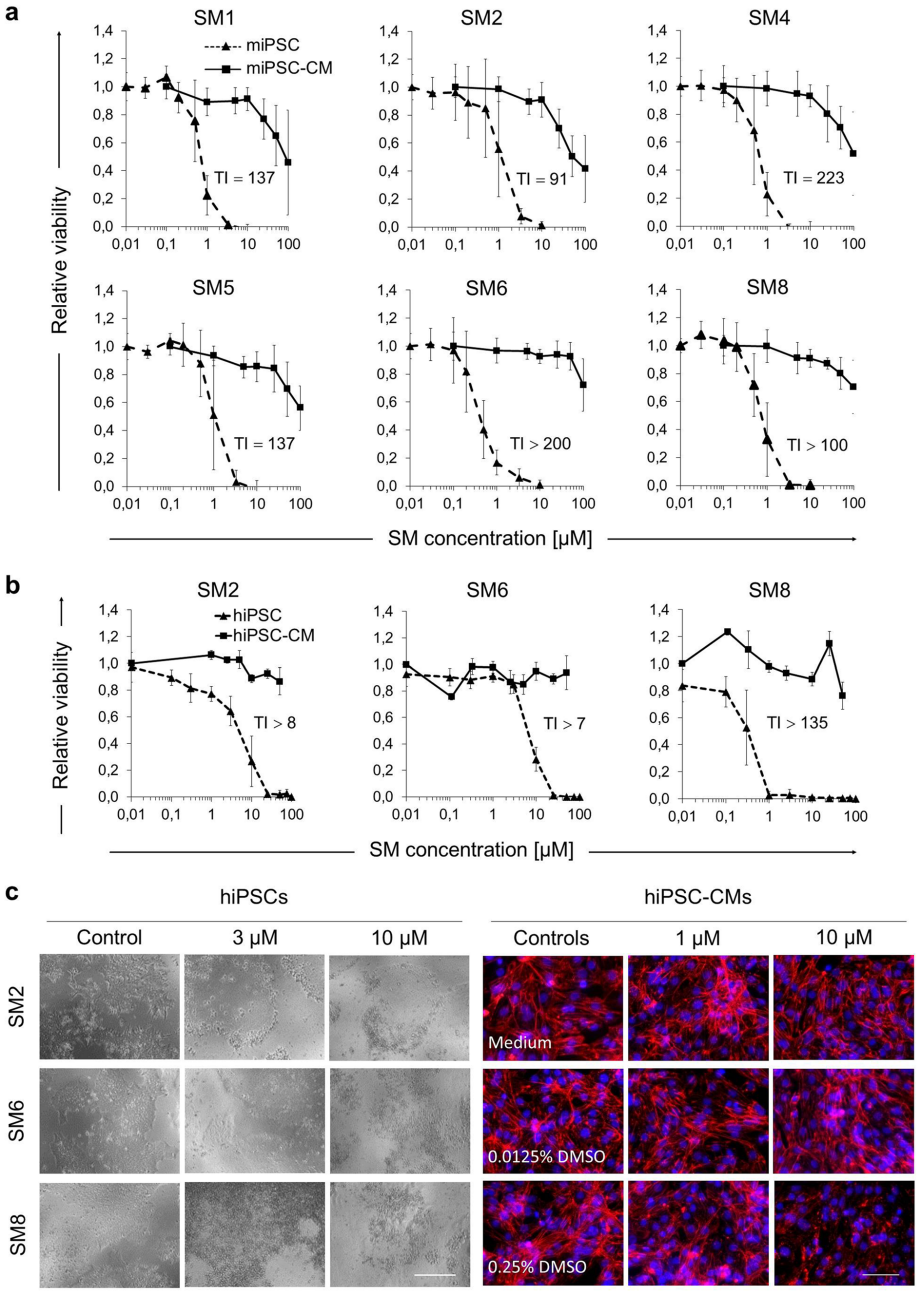
Cytotoxic effects of selected SMs on undifferentiated murine iPSCs and purified murine and human iPSC-CMs. (**a**) Dose response curves for cytotoxicity of indicated SMs on miPSCs and miPSC-CMs after 48 h of treatment with various SM concentrations. Results are shown as relative values compared to 0.05% DMSO treated cells (mean ± SD). Data for miPSCs were pooled from two (SM1, SM4, SM5), three (SM2, SM8) or six (SM6) independent experiments with 4 replicates analyzed in each. Analyses with miPSC-CMs were performed in three independent experiments for each SM. Therapeutic index (TI) was calculated by dividing the IC50 of miPSC-CMs by the IC50 of miPSCs. (**b**) Dose response curves for cytotoxicity of indicated SMs on human iPSCs (NP0040 cell line) and human iPSC-CMs (day 46 of differentiation) after 48 h of treatment with various SM concentrations. Results are shown as relative values compared to 0.05% DMSO treated cells (mean ± SD). Data for hiPSCs-CMs were pooled from two independent experiments with 4 replicates analyzed in each. Therapeutic index (TI) was calculated by dividing the IC50 of hiPSC-CMs by the IC50 of hiPSCs. (**c**) Representative images of NP0040 hiPSCs (bright-field) and hiPSC-CMs (immunofluorescence) after 48 h treatment with controls and various concentrations of indicated SMs. At the end of the treatment, hiPSC-CMs were fixed with 3% PFA and stained with antibodies against sarcomeric α-actinin (red). Nuclei were counterstained with Hoechst 33342 (blue). Images shown are digitally magnified insets from the originals taken with the 20 × objective on Axiovert 200 M microscope. Scale bars: 50 μm. See also Supplementary Figs. S2, S3 and S4.

Since CMs are one of the most widely used PSC-derivatives, we next sought to determine whether the SMs identified to be harmful to PSCs would also be toxic for CMs derived from them. Comparison of cytotoxic responses revealed that none of the tested SMs affected the viability of purified miPSC-CMs even when used at concentrations that were highly toxic to undifferentiated iPSCs (Fig. 2a and Supplementary Fig. S4a). Prolonged treatment (72 h) with 10 µM of SM2, SM5 and SM6 resulted in only a minor decrease of CM viability by, respectively, 18.5 ± 7.1% (p < 0.01), 14.1 ± 13.8% (p > 0.05) and 11.0 ± 13.2% (p > 0.05) (Supplementary Fig. S4b). However, 72 h treatment with 10 µM of SM1, SM4 and SM8, which have identical constitution but are stereoisomeric to one another, decreased the miPSC-CM viability by an average of 79.8 ± 14.7%. Additional analyses showed that the IC_50_ values of SM1, SM2, SM4, SM5, SM6 and SM8 for miPSC-CMs were, respectively, 137-, 91-, 223-, 137-, > 200- and > 100-fold higher than those for iPSCs, further demonstrating highly selective toxicity of these compounds for undifferentiated mPSCs (Fig. 2a). Cytotoxicity assays performed with hiPSC-CMs demonstrated that the IC_50_ values of SM2, SM6 and SM8 were more than eight-, seven- and 135-fold higher than those for undifferentiated hiPSCs (Fig. 2b), suggesting that some SMs might exhibit lower selectivity towards human than murine PSCs.

### Salicylic diamines exert no or only minor and reversible side-effects on iPSC-CMs

**Figure 3:**
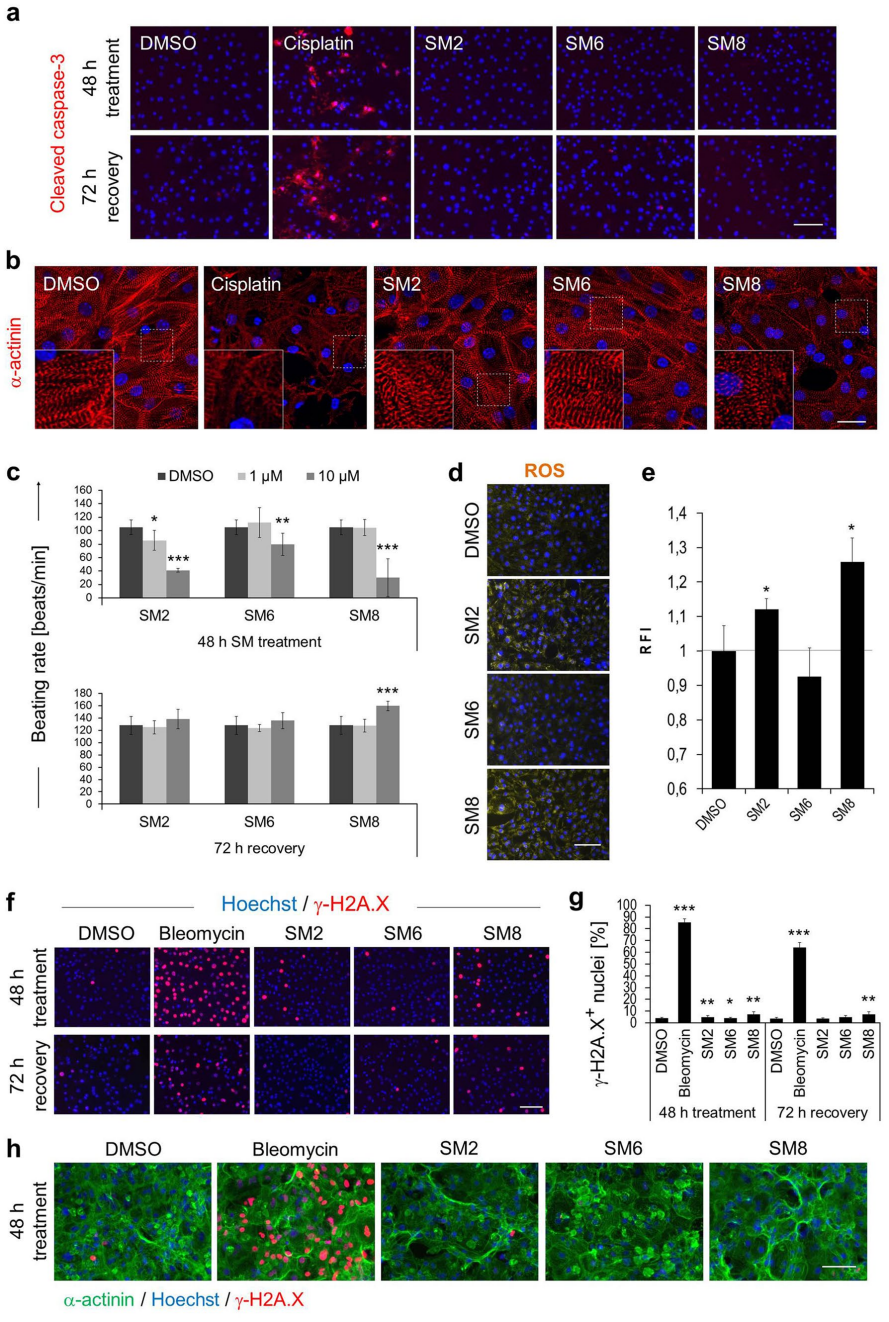
Effect of SM2, SM6 and SM8 on iPSC-CMs. (**a**, **b**) Representative images of active caspase-3 (**a**, red) and sarcomeric α-actinin (**b**, red) in αPIG-AT25-iPSC-derived CMs treated for 48 h with 10 µM of indicated SMs (in **a**, **b**) and after 72 h of recovery (in **a**). Insets in b: magnified views of boxed areas. (**c**) CM beating rates after 48 h of SM treatment and 72 h of subsequent recovery (mean ± SD; N = 8 pooled from two independent experiments). (**d**, **e**) ROS-levels in CMs after 48 h of 10 µM SM-treatment were visualized by fluorescence microscopy using CellROX reagent (**d**, yellow) and quantified by Image J (**e**). Data are presented as mean ± SD (n = 3). RFI: relative fluorescence intensity. Similar results were obtained in one additional independent experiment. (**f**, **g**) Representative images of miPSC-CMs (**f**) stained for a DNA damage marker γ-H2A.X (red) after 48 h of treatment with 10 µM of indicated SMs and 72 h of recovery (**f**). Quantification of γ-H2A.X positive nuclei in this experiment is shown in g (mean ± SD; n = 5 wells; on average, a total of 770 nuclei were scored per group). Similar results were obtained in one additional independent experiment. (**h**) ICC staining of α-actinin (green) and γ-H2A.X (red) after 48 h treatment of Cor.4U hiPSC-CMs provided by Axiogenesis with 10 µM SM2, 3.3 µM SM6 and 0.5 µM SM8. Negative controls in all experiments included 0.05% DMSO. Positive controls included 75 µg/ml cisplatin (A, B) or 20 µg/ml bleomycin (F, G). Nuclei in panels (**a**, **b**, **d**, **f** and **h**) were stained in blue with Hoechst 33,342. Scale bars: 25 µm (**a**), 100 µm (**b**, **d**, **f**, **h**). * p < 0.05, ** p < 0.01, *** p < 0.001. See also Supplementary Figs. S4, S5 and S6.

Although the above data show that salicylic diamines are selectively toxic to PSCs but not to CMs, these compounds might still exert subtle adverse effects on CM physiology which may diminish their functionality and hamper downstream applications. To evaluate the potential side-effects of SM2, SM6 and SM8 in miPSC-CMs we first assessed the apoptosis rate by detecting the active form of caspase-3. A high proportion of apoptotic CMs was detected only in the control group treated for 48 h with cisplatin but not in CMs treated with 10 µM of SM2, SM6 or SM8 (Fig. 3a). The rate of apoptosis was also not increased in CMs that were cultured for 72 h without SMs following the initial 48 h exposure to these drugs, indicating that they have no delayed adverse effects on CM viability. Morphology and sarcomeric integrity of mPSC-CMs were not compromised by treatment with these SMs as revealed by confocal microscopy of α-actinin-stained cells (Fig. 3b). Microscopic examination of hiPSC-CMs revealed that their viability was not affected by 48 h treatment with SM2 and SM6 at concentrations up to 25 µM that were toxic to undifferentiated hiPSCs (Fig. 2c, Supplementary Fig. S4d). However, treatment of hiPSC-CMs with 50 µM SM6 or 10 SM8 visibly reduced the hiPSC-CM confluency and affected the cell morphology (Fig. 2c and Supplementary Fig. S4d).

We next assessed the effect of SM2, SM6 or SM8 on the beating rate of CMs. Compared to DMSO-treated cells, beating rate of miPSC-CMs was significantly decreased after 48 h treatment with 10 µM of each of these drugs (Fig. 3c, upper panel). Yet, after a recovery period of 72 h after SM treatment, SM2- and SM6-treated CMs fully regained their initial beating frequencies while SM8-treated CMs exhibited 25 ± 6% higher beating rates than control CMs (p < 0.001; Fig. 3c, lower panel), suggesting that initial side-effects can be fully reversed after subsequent culture without drugs. Spontaneous beating activity in cultures of hiPSC-CMs was only reduced or abolished when they were treated with high concentrations of SM2 (50 µM) or SM6 (25 µM) but SM8 blocked CM contractions already at the concentration of 2.5 µM without affecting the CM viability (Supplementary Fig. S4c), which is in agreement with the observation that SM8 is more toxic to murine iPSC-CMs than SM2 or SM6 (see Supplementary Fig. S4b).

Various drugs exert cytotoxicity by inducing oxidative stress. Analysis of reactive oxygen species (ROS) levels in miPSC-CMs after exposure to SM2, SM6 or SM8 using the fluorogenic ROS probe showed that ROS levels were not increased in CMs treated with 1 µM of any of these SMs (data not shown) or with 10 µM of SM6. However, ROS levels were significantly increased when CMs were treated with 10 µM of SM2 or SM8 (Fig. 3d,e), suggesting that these two SMs are more prone to induce side-effects in mPSC-CMs than SM6.

Since a variety of drugs cause DNA damage which lead to pleiotropic cellular responses detrimental to normal cell functions, we next sought to determine whether SMs exert genotoxic effects in PSC-CMs. Bleomycin, which served as positive control, efficiently induced DNA double-strand breaks in more than 80% of miPSC-CMs as indicated by increased phosphorylation of the histone H2AX (γ-H2A.X). In contrast, treatment with 10 µM of SM2, SM6 or SM8 increased the fraction of γ-H2A.X-positive CMs only marginally above the background value found in DMSO-treated control cells (Fig. 3f,g). SM8 increased the fraction of γ-H2A.X-positive cells from 4.2 ± 0.7% found in the control group to 9.8 ± 3% (p < 0.01). This effect persisted even after subsequent cultivation of CMs for 72 h in the absence of this drug (Fig. 3f,g). Compared to SM8, SM2 and SM6 caused DNA damage in a smaller proportion of CMs immediately after 48 h treatment but after 72 h of recovery in the absence of these drugs the fraction of γ-H2A.X-positive nuclei returned to background levels (Fig. 3f,g). In hiPSC-CMs, SM2, SM6 or SM8 exerted no significant DNA-damage at hiPSC-toxic concentrations (Fig. 3h). Collectively, these findings indicate that PSC-CMs tolerate SMs fairly well and that SM6 might be the most promising PSC-eliminating compound because it exerted high toxicity on PSCs but only transient and mild effects, if any, on pure CMs.

### Salicylic diamines decrease oxygen consumption rate (OCR) in iPSCs

**Figure 4:**
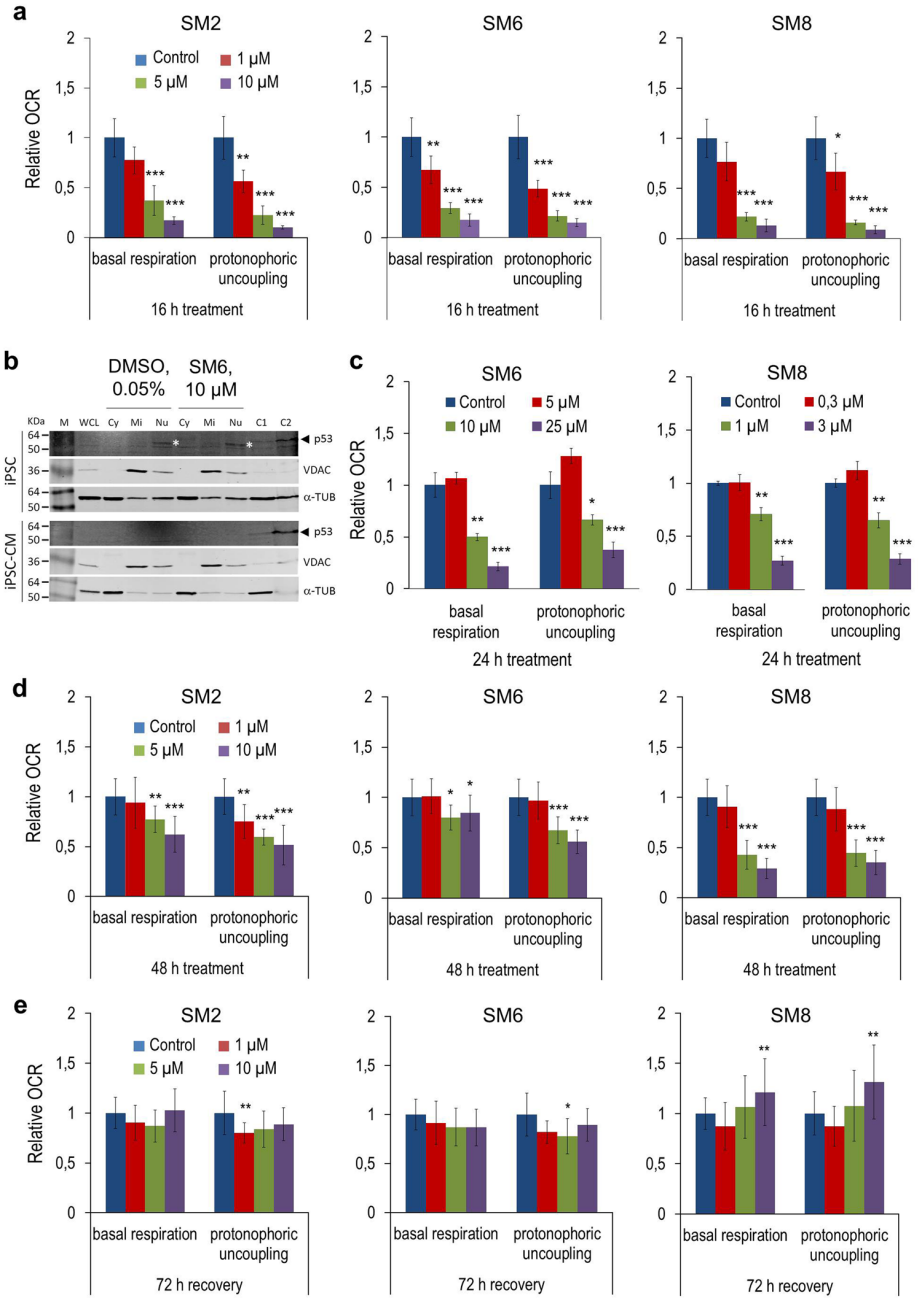
Salicylic diamines inhibit basal respiration and maximal respiratory capacity to a different extent in miPSCs, hiPSCs and miPSC-CMs. (**a**, **c**–**e**) Basal respiration and OCR response to FCCP-induced (1 µM) protonophoric uncoupling in undifferentiated αPIG-AT25 miPSCs after 16 h of SM treatment (**a**), in undifferentiated NP0040 hiPSCs after 24 h of treatment (**c**) and in purified miPSC-CMs after 48 h of SM treatment (**d**) as well as after 72 h of recovery following treatment (**e**). Data are shown as relative values compared to 0.05% DMSO-treated control cells and are presented as mean ± SD of n = 3 measurements from one experiment in (**a** and **c**), and n = 6 measurements pooled from two independent experiments in d and e. Each measurement was performed with 7 replicates per group. See also Supplementary Fig. S5. (**b**) Immunoblot analysis. miPSCs and miPSC-CMs were treated with 10 µM SM6 or vehicle (0.05% DMSO) for 8 h and then fractionated into cytoplasmic (Cy), mitochondrial (Mi) and nuclear (Nu) fractions. Levels of p53, cytosolic marker α-tubulin (α-TUB), and mitochondrial marker voltage-dependent anion channel (VDAC) were determined in each fraction and compared with those from whole cell lysates (WCL) by immunoblotting. C1 and C2 indicate control WCLs prepared from, respectively, human HEK293 and HT29 cells in the panel for iPSCs, and HEK293 and COS9 cells in the panel for iPSC-CMs. They served as positive controls for p53. Molecular weight of human p53 is slightly higher than that of murine p53 which explains different positions of this protein in murine iPSC and iPSC-CM (*) and human control samples in C1 and C2 lanes (◄). Full-length immunoblots of all analyses are shown in Supplementary Figs. S8, S9 and S10. *KDa* kilodalton, *M* protein marker.

In order to investigate the mechanism of SM-toxicity, we analyzed the effect of SMs on mitochondrial OCR in murine and human iPSCs and miPSC-CMs, because these cell types markedly differ in the activity of mitochondria-mediated apoptotic and metabolic pathways^49^. In these analyses, miPSCs were incubated with SMs for only 16 h in order to enable the assessment of oxygen consumption in still viable cells. Under these conditions, SM2, SM6 or SM8 used at the concentration of 1 µM inhibited basal respiration and maximal cellular respiration (assessed in the presence of the uncoupling agent FCCP) by about 20–50%. However, no significant effects on OCR were seen at this concentration in the presence of ATP-synthase inhibitor oligomycin, which was added to differentiate the ATP-linked respiration from the proton leak or in the presence of antimycin A and rotenone which served to assess the extent of non-mitochondrial respiration (Fig. 4a and Supplementary Fig. S5a). At 5 and 10 µM concentrations all three SMs reduced OCR in miPSCs to almost undetectable levels (Supplementary Fig. S5a), which was most likely responsible for mPSC killing after longer exposure times. The mitochondrial p53 accumulation didn’t seem to play a role in apoptosis induced by SM6 in miPSCs (Fig. 4b) as it was recently shown for the inhibitors of survivin in hPSCs by Lee et al.^39^.

Human iPSCs were also susceptible to inhibition of OCR by SM6 and SM8 but to a somewhat lesser extent than miPSCs. While OCR in miPSCs was almost completely abolished by 16 h exposure to 5 µM SM6, no effect was seen at this concentration in hiPSCs after 24 h treatment. Significant inhibition of OCR in hiPSCs was achieved only with 10 and 25 µM SM6. In contrast, SM8 appeared to exert similar inhibitory effects on OCR in murine and human iPSCs (Fig. 4c and Supplementary Fig. S5b). This observation is consistent with the comparable IC_50_ values of SM8 for murine and human PSCs and suggests that SM6 and SM8 might use different mechanisms to induce cell death in these cells.

In contrast to miPSCs, SMs measurably inhibited OCR in miPSC-CMs when the treatment was prolonged to 48 h and only when SMs were used at concentrations of 5 or 10 µM (Fig. 4d and Supplementary Fig. S5c). Under these conditions, the strongest inhibition of OCR in CMs was observed with 10 µM SM8 (average decrease by 67 ± 13% compared to DMSO-treated control cells, p < 0.001) and the lowest inhibition with 10 µM SM6 (average decrease by 30 ± 24% compared to control cells, p < 0.05) (Fig. 4d and Supplementary Fig. S5c). Importantly, the diminished OCR in miPSC-CMs completely returned to control levels after 72 h of recovery post SM2 and SM6 treatment or became even higher in CMs treated with 10 µM of SM8 than in control cells (1.3 ± 0.4-fold, p < 0.01) (Fig. 4e and Supplementary Fig. S5d). These findings demonstrate that SMs exert their cytotoxic effects by inhibiting mitochondrial ATP production in PSCs and that in murine CMs these effects are mild, transient and unable to compromise their viability under these conditions.

### SM6 efficiently eliminates residual mPSCs in partially purified cardiac clusters

**Figure 5:**
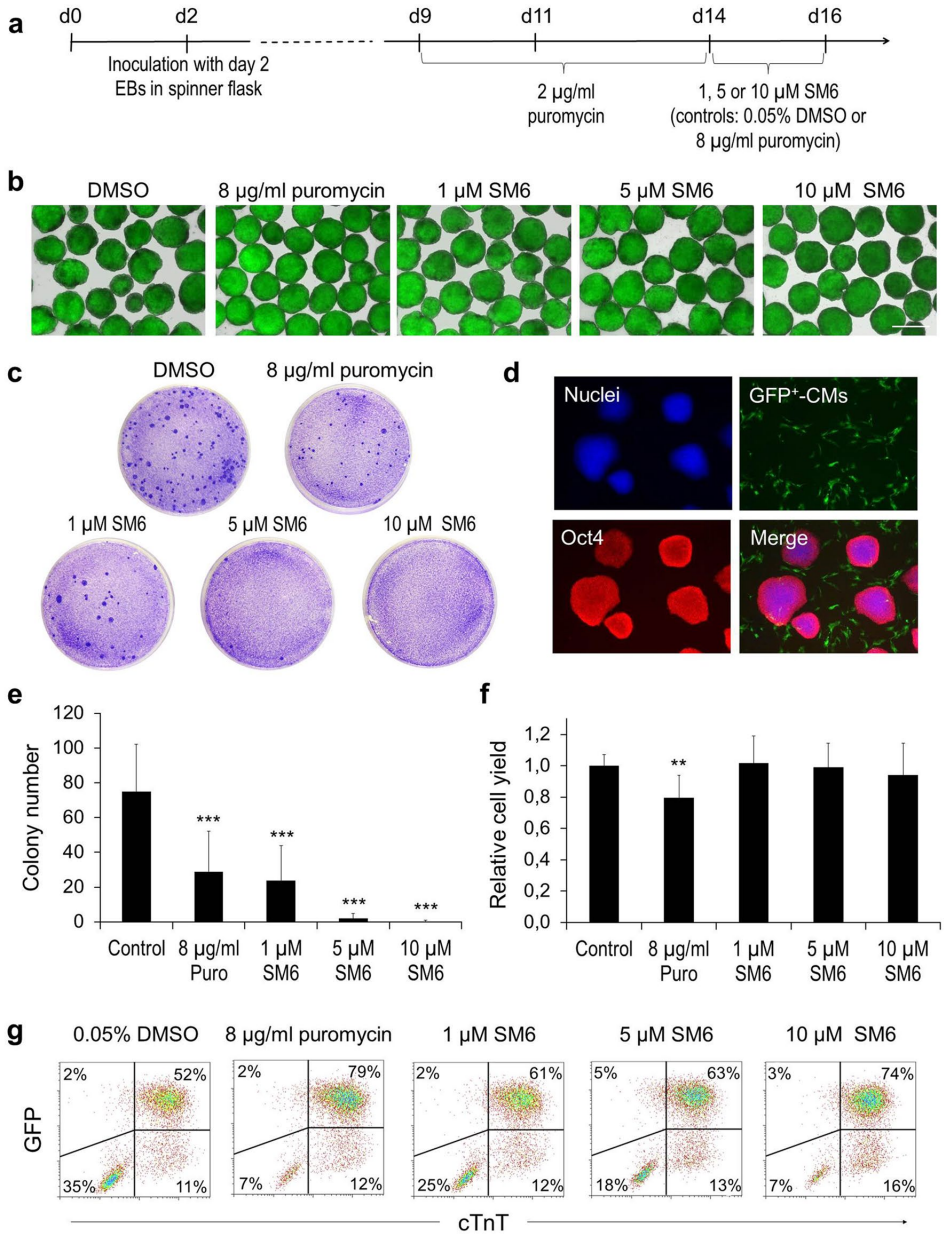
SM6 selectively eliminates undifferentiated PSCs in partially purified miPSC-CMs generated in suspension cultures as cardiac clusters. (**a**) Time course of αPIG-AT25 miPSC cardiogenic differentiation and drug treatment. (**b**) Representative bright field and GFP-fluorescence images (overlay) of miPSC-derived GFP-expressing cardiac clusters from day 16 of differentiation after 2 day treatment with indicated drugs. Scale bar: 100 µm. (**c**) Representative images of crystal-violet-stained PSC-colonies (dark blue spots) formed after growing 2 × 10^5^ cells derived from day 16 cardiac clusters in one 6 cm plate for 7–10 days in mPSC culture conditions. (**d**) Oct4 expression (red) confirms the PSC identity of colonies detected by crystal violet in panel C. (**e**) Quantification of PSC-colonies per 2 × 10^5^ cells/plate seeded as described in panel C (mean ± SD; n = 19 from five independent experiments). *** p < 0.001. (**f**) Total yield of cells harvested from day 16 cardiac clusters after treatment with indicated drugs. Results are shown as relative values compared to DMSO-treated control (mean ± SD; n = 9 from five independent experiments). ** p < 0.01. (**g**) Flow cytometric analysis of GFP-expressing day 16 CMs treated with indicated reagents and stained for cardiac troponin T (cTnT) in one representative differentiation experiment. See also Supplementary Fig. S7.

In previous experiments, the effect of SMs on iPSCs and CMs was tested separately for each cell type. Next, we sought to determine whether SM6 is also able to selectively eliminate PSCs in mixed cultures with CMs without affecting CM viability. In this protocol (Fig. 5a), CMs in differentiating αPIG-AT25 miPSC suspension cultures were first purified for five days with low concentration of puromycin (2 µg/ml). The resulting cardiac clusters containing a residual fraction of PSCs were then treated for two additional days with 1, 5 or 10 µM SM6, or with 0.05% DMSO or 8 µg/ml puromycin which served as negative and positive controls for CM purification, respectively^48^. The efficiency of PSC elimination in each of these groups was then determined by PSC-colony formation assay.

Microscopic and electrophysiological analyses revealed that the morphology, viability and action potential properties of cardiac clusters were not significantly compromised by SM6 at any of the applied concentrations (Fig. 5b, Supplementary Fig. S6a–g and Supplementary Results). Quantification of the residual miPSCs in SM6-treated cardiac clusters showed that 1 µM SM6 reduced the number of miPSCs to the same extent as 8 µg/ml puromycin (Fig. 5c,e). The application of SM6 at 5 or 10 µM led to even stronger reduction of contaminating miPSCs yielding per 2 × 10^5^ CMs, respectively, only 2.0 ± 2.9 and 0.4 ± 0.6 PSC colonies, compared to 75 ± 27 colonies in the DMSO group and 29 ± 23 colonies in the puromycin group (Fig. 5e). Importantly, the total cell yield in cardiac clusters in the SM6 group was similar to the cell yield in the DMSO group and higher than that in the puromycin group (Fig. 5f). Flow cytometry showed that SM6 was as potent as puromycin in reducing the PSC-containing fraction of GFP^-^/cTnT^-^ non-CMs in these partially purified cardiac clusters (Fig. 5g). This fraction was diminished from 35% in the DMSO group to about 7% in the groups treated with 10 µM SM6 or with 8 µg/ml puromycin (Fig. 5g). Accordingly, the fraction of cTnT^+^ CMs increased from 63% of all viable cells in the DMSO group to 91% and 90% in the puromycin- and SM6-treated groups, respectively (Fig. 5g). Similar PSC-eliminating and CM-enriching effects of SM6 were reproduced with the murine transgenic ESC line αPIG44-D3 which was differentiated in large-scale suspension culture in spinner flasks (Supplementary Results and Supplementary Fig. S7), demonstrating a broad validity of these results.

### Attenuation of teratoma formation by SM6

**Figure 6:**
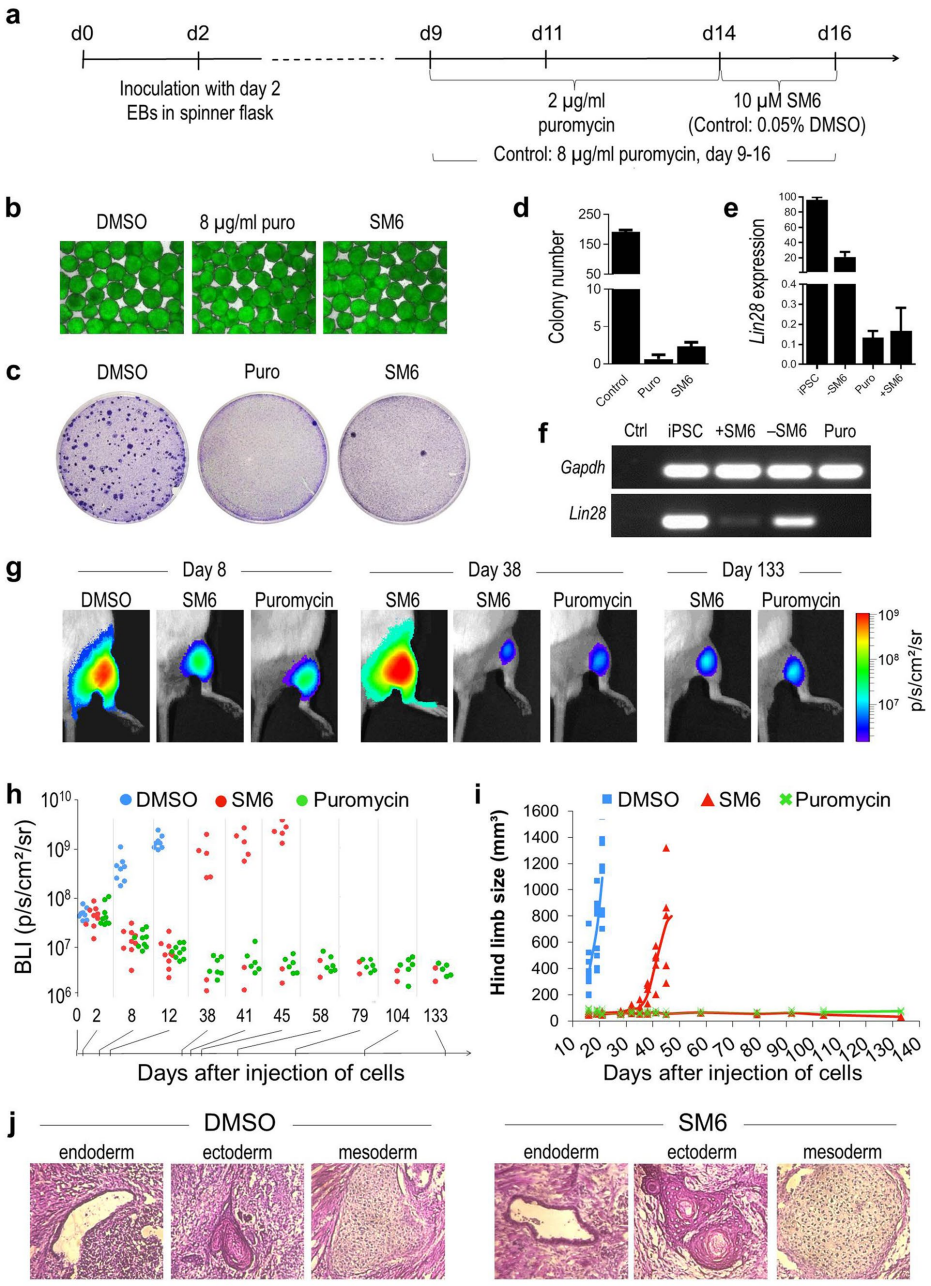
SM6 reduces the risk of teratoma formation from residual PSCs in miPSC-CM preparations. (**a**) Time course of cardiac differentiation of firefly luciferase (FLuc)-expressing αPIG-AT25 miPSCs and drug treatment schedule. (**b**) Representative images of day 16 cardiac clusters after treatment with indicated drugs. Scale bar: 100 µm. (**c**, **d**) Determination of miPSC contamination levels in control and drug treated day 16 cardiac clusters by crystal-violet staining. PSC-colonies were identified as dark blue spots (c) and quantified (d) after growing 2 × 10^5^ cells derived from dissociated clusters for 7–10 days under mPSC culture conditions. The number of PSC-colonies found in each group is presented as mean ± SD of n = 3 plates. (**e**, **f**) Expression of *Lin28* mRNA in αPIG-AT25 miPSCs and day 16 cardiac clusters after 48 h treatment with indicated drugs. RNA was isolated and RT-qPCR (**e**) and semi-quantitative RT-PCR (**f**) were performed. Gene expression levels in e were plotted relative to *Lin28* expression in miPSCs. GAPDH was used as a reference gene. (**g**) Representative BL images of mice taken on day 8, 38 and 133 after injection into the right hind limb of 1 × 10^6^ FLuc-expressing cells dissociated from day 16 cardiac clusters after treatment with indicated drugs. (**h**, **i**) BL signal intensities (**h**) and hind limb volumes (**i**) measured at the indicated time points after miPSC-CM transplantation. Each data point represents one animal (n = 6–9 mice per group). (**j**) Representative H&E-stained images of teratomas formed from DMSO- and SM6-treated miPSC-CMs in mice on day 21 and 42 after transplantation, respectively.

In order to determine whether the dramatic SM6-mediated reduction in residual miPSC levels in mPSC-CM preparations would eliminate their tumorigenicity in vivo, CMs were prepared following the protocol depicted in Fig. 6a from a transgenic miPSC line constitutively expressing firefly luciferase on a αPIG-AT25 genetic background (FLuc-αPIG-AT25)^50^. This enabled preparation of CMs at various degrees of purity and non-invasive in vivo monitoring of transplanted cells by bioluminescence imaging (BLI). Similarly to experiments described above, the number of residual PSCs in these CM preparations was dramatically decreased from 192 ± 7 PSC colonies per 2 × 10^5^ CMs in DMSO control group to only 2.3 ± 0.6 PSC colonies in 10 µM SM6 group and 0.7 ± 0.6 PSC colonies in 8 µg/ml puromycin group (Fig. 6b–d). Efficient elimination of residual PSCs was also confirmed by a dramatic reduction of *Lin28* mRNA levels (Fig. 6e,f) as well as by decrease of Oct4 transcript levels and of SSEA-1- expressing iPSCs (data not shown) in cardiac clusters treated with puromycin and SM6. Injection of 1 × 10^6^ CMs from the DMSO control group into the hind limb muscle of immunodeficient NSG mice resulted in the development of teratoma in all 9 transplanted mice. Significantly, increased BLI signal was detectable already on day 8 post transplantation and the increasing teratoma size necessitated termination of the experiment in this group of animals on day 21 after cell transplantation (Fig. 6g–i). In contrast, SM6-treated CMs produced teratomas in 5 out of 7 mice but they emerged with a significant delay on day 28–32 after cell injection as determined by palpation and quantification of tumor size. The remaining 2 animals in the SM6 group as well as all 7 animals in the puromycin group formed no teratomas but still retained the BLI signal until as late as 19 weeks post cell transplantation. Histological analyses of teratomas in the DMSO and SM6 group showed the presence of tissues representing derivatives of all three primary germ layers confirming their origin from contaminating iPSCs (Fig. 6j).

### Enrichment of CMs by SM6 treatment alone

**Figure 7:**
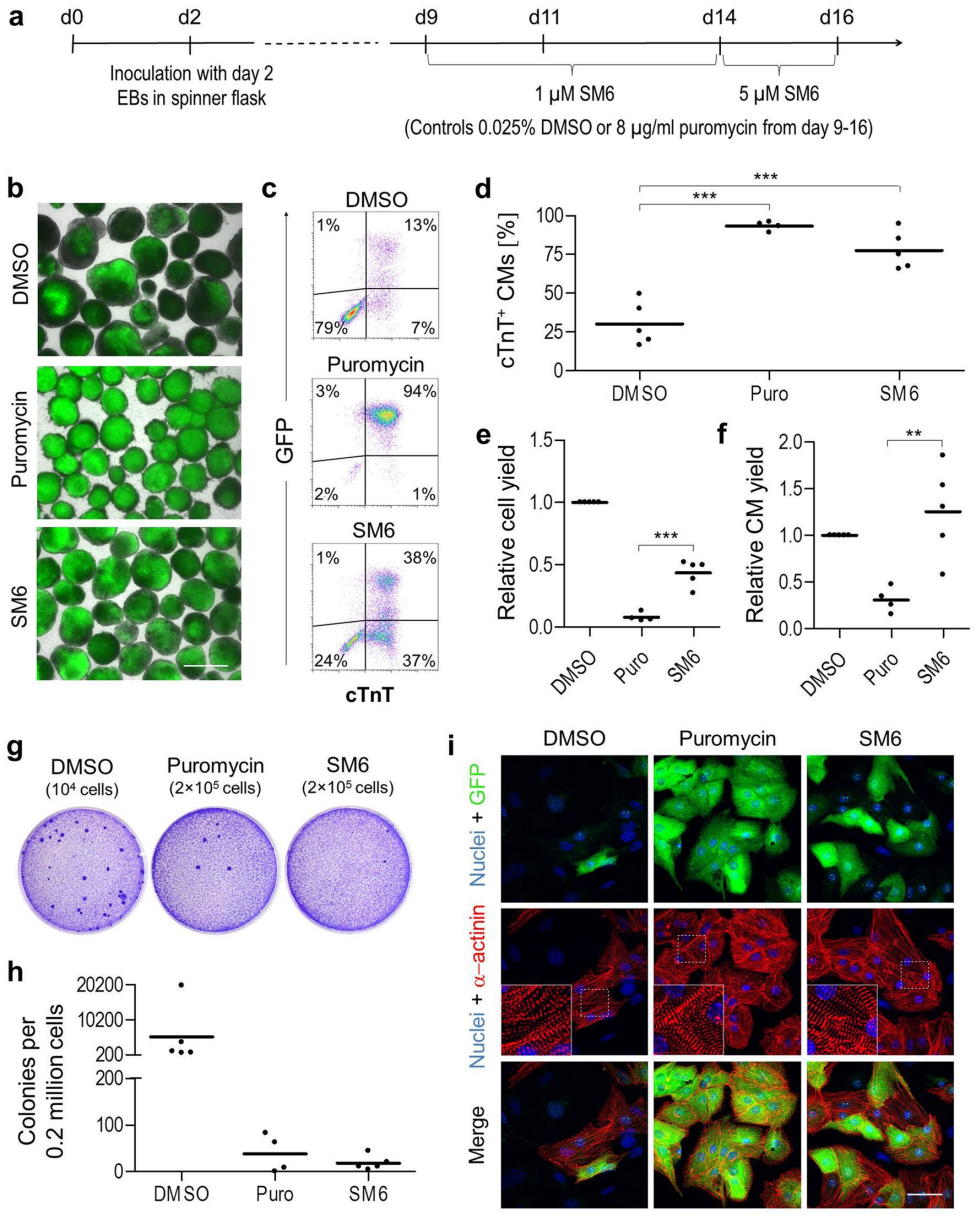
Selective elimination of PSCs and enrichment of CMs with SM6 alone. (**a**) Time course of cardiac differentiation of αPIG-AT25 miPSCs and drug treatment schedule. (**b**) Representative bright field and GFP-fluorescence images (overlay) of cell aggregates on day 16 of differentiation. Scale bar: 100 µm. (**c**, **d**) Flow cytometric analysis (**c**) and quantification (**d**) of CM purity in dissociated day 16 clusters after treatment with indicated drugs. CMs were identified based on expression of αMHC-driven GFP and endogenous cTnT. Each data point in d represents results from one independent experiment (n = 4–5). (**e**, **f**) Yields of all cells (**e**) and cTnT^+^ CMs (**f**) in puromycin- and SM6-treated day 16 cardiac clusters relative to DMSO-treated controls shown as individual data points from 4–5 independent experiments. (**g**–**h**) Representative images (**g**) and quantification (**h**) of crystal-violet-stained PSC colonies formed after plating the indicated number of cells derived from day 16 clusters in each group onto one 6 cm plate and growing for 7 days under mPSC culture conditions. Individual data points represent average number of PSC-colonies detected in each of 4–5 independent differentiation experiments. (**i**) Representative confocal microscopic images of α-actinin-stained CMs derived from day 16 clusters in respective experimental groups. Scale bar: 50 µm. Insets: enlarged views of boxed areas. Horizontal lines in panels (**d**, **e**, **f** and **h**) indicate means. **p < 0.01, ***p < 0.001.

In order to determine whether it is possible to entirely replace puromycin by SM6 in the process of CM purification and thus circumvent the need for genetic manipulation of PSCs, we developed a stepwise CM purification protocol with SM6 at concentrations high enough to enable elimination of undifferentiated miPSCs and other non-cardiac differentiated cells but still low enough to prevent cytotoxicity towards CMs. In this protocol, embryoid bodies (EBs) were treated with 1 µM SM6 from day 9 to day 14 of differentiation which was then followed by incubation with 5 µM SM6 from day 14 until day 16 of differentiation (Fig. 7a). Fluorescence microscopic analysis of cell aggregates collected on day 16 of this protocol revealed that SM6 enriched the CM content in these clusters as estimated by the size of GFP-positive areas (Fig. 7b). Flow cytometric quantification of genetically engineered (GFP) and endogenous (cTnT) cardiac markers in dissociated CMs showed that DMSO-treated cell aggregates contained 79% non-CMs and only 21% of cTnT^+^ CMs (Fig. 7c). Among all cTnT^+^ CMs, 13% were GFP^+^ and 7% were GFP^-^ indicating that αMHC-driven GFP does not demarcate all PSC-derived CMs but only a subpopulation of these cells in which this promotor is active at this developmental time point (Fig. 7c). Purification with 8 μg/ml puromycin yielded cTnT^+^ CMs at the expected purity of 95% and almost entirely eliminated the GFP^−^/cTnT^−^ non-CMs as well as the GFP^-^ subpopulation of cTnT^+^ CMs that was present in the SM6 group (Fig. 7c). Treatment with SM6 also led to a significant enrichment of cTnT^+^ CMs (75%) but the GFP^-^ fraction of cTnT^+^ CMs was completely preserved compared to the puromycin group and was approximately of the same size (37% of all cells in the lower right quadrant) as the fraction of GFP^+^/cTnT^+^ CMs (38% of all cells in the upper right quadrant) which is more than five times larger than the GFP^-^ fraction of cTnT^+^ CMs in the DMSO group (Fig. 7c). In addition, the fraction of GFP^−^/cTnT^−^ non-CMs was much lower (24% of all cells) than in the DMSO group (79% of all cells) but higher compared to the puromycin group (2%) indicating that SM6 under this experimental conditions eliminated some but not all non-cardiac differentiated cell types (Fig. 7c).

Flow cytometric quantification of the cTnT^+^ CM population in five independent experiments revealed that both puromycin and SM6 enriched CM purity by about 2.5- to threefold compared to DMSO-treated cells (Fig. 7d). However, the CM purity was lower in SM6-treated (78 ± 12%) compared to the puromycin-treated cells (93 ± 3%) which is in agreement with the observation above that SM6 was not toxic to a subset of cTnT^-^ non-CMs (Fig. 7d). Compared to DMSO control cells, CM purification with puromycin resulted in a strong 92 ± 4% decrease in the total cell yield after dissociation of cell aggregates at day 16 of differentiation while in the SM6 group the total cell yield was reduced only by 57 ± 10% (Fig. 7e). However, the treatment with SM6 resulted in a 2.7 ± 1.6-fold higher yield of cTnT^+^ CMs compared to puromycin treatment (p < 0.01, Fig. 7f). In three out of five experiments the CM yield was even 31–86% higher in the SM6 group compared to the DMSO control group indicating that CMs still may proliferate in the presence of SM6 (Fig. 7f).

We next set out to determine if the preservation of GFP^−^/cTnT^−^ cell population in SM6-treated group is due to inability of this drug to completely eliminate undifferentiated PSCs and/or due to a selection of an additional non-cardiac differentiated cell population(s). The colony formation assay revealed that SM6 led to a strong reduction of contaminating colony-forming PSCs by 99 ± 1% which was comparable to the standard procedure using puromycin (98 ± 4%, Fig. 7g,h). Immunocytochemical analyses demonstrated that CMs produced both by SM6 and control procedures were structurally intact and possessed well-defined sarcomeric structures and that the contamination level by α-actinin-negative non-CMs was the highest in the DMSO group and non-existent in the puromycin group (Fig. 7i). This analysis also confirmed the presence of a subpopulation of α-actinin-positive but GFP-negative CMs in the SM6 and DMSO but not in the puromycin group as already shown by flow cytometry (Fig. 7i). These data demonstrate the value of SM6 not only for elimination of PSCs from differentiated cultures without requirement for genetic selection but also for enrichment of more heterogeneous CM populations which are otherwise eliminated by standard genetic selection procedures.

## Discussion

Residual PSCs in a PSC-derived cell population represent a major obstacle for the clinical translation of stem cell research. Various strategies have been reported for the removal of contaminating PSCs ranging from genetic^18,27–34^ and immunologic^9–14^ selection methods, metabolic^23–26^ or phototoxic ablation strategies^18,51^ to PSC-selective cytotoxic compounds^35–42^. Among them, cytotoxic SMs represent a very attractive approach because they offer simplicity, accessibility, scalability and flexibility in combinatorial application at specific times, durations and doses in various platforms without the need for additional cell processing or genetic modification.

In this study, we describe salicylic diamines as novel compounds that are highly toxic to several murine and human PSC lines at low micromolar concentrations. At these concentrations, the most potent and selective SMs did not affect the viability of PSC-CMs. Detrimental effects observed in PSC-CMs were caused only at higher SM concentrations and after longer periods of treatment, and these side-effects were fully reversible after a short recovery period in miPSC-CMs. The toxicity of SM2 and SM6 appeared to be higher against murine than human PSCs while SM8 exhibited comparable IC_50_ values for both groups. The diamino cyclohexane-based core structure of these SMs with its *ortho*-hydroxybenzylamine moieties was essential for the PSC killing. The results showed that substituents added to the amino groups or to the benzylic carbon significantly reduced the cytotoxicity of the salicylic diamines against PSCs. Moreover, dose–response experiments with murine PSCs with compounds structurally related to the most active and selective compound SM6 demonstrated that the activity was dependent on the proper constitution of this molecule. Altering the position or removal of the phenolic hydroxyl groups erased the cytotoxic effect of SM6 on PSCs, indicating a defined mechanism of action in PSCs.

The most likely mechanism underlying selective toxicity of salicylic diamines SM6 and SM8 towards murine and human iPSCs is a strong inhibition of mitochondrial respiration and this effect correlated with their PSC-cytotoxicity. The differential susceptibility of human and murine iPSCs to SM6 is most likely due to different developmental stages^52^ and metabolic states in which they exist^53–55^. The conventional hPSCs are in a primed state and are essentially glycolytic, while mPSCs exist in a naïve pluripotent state and use both glycolysis and OXPHOS pathways in their energy production^56^. Since ATP production from OXPHOS in primed hiPSCs is less critical for their survival, they appear to be less susceptible to toxic effects of SM6 than miPSCs. Interestingly, SM8 was similarly potent in inducing toxicity and inhibiting OCR in murine and human iPSCs suggesting that this compound might operate with a different mechanism of action than SM6.

The exact mechanism by which salicylic diamines inhibit OCR and cause toxicity in PSCs is unclear and remains to be elucidated in future studies. These SMs could lead to the killing of PSCs by targeting specific electron transport chain complexes leading to ATP depletion, mitochondrial membrane potential reduction and/or oxidative damage induction, as was shown for different OXPHOS inhibitors in cancer cells reliant on OXPHOS^57,58^. Subsequent cytochrome c release from damaged mitochondria into the cytoplasm could activate the caspase cascade and induce apoptosis. It is also possible that SM-mediated energy depletion leads to activation of AMP-activated protein kinase (AMPK) which serves as a key regulator of energy production pathways in many cell types. AMPK activation in CMs increases their survival by restoring energy homeostasis and inducing cardioprotective effects^59^. However, activation of AMPK by the OXPHOS inhibitor metformin decreases reprogramming efficiency of murine and human iPSCs^60^. Therefore, it is possible that the selective toxicity of salicylic diamines to PSCs and CMs is based on their differential regulation of AMPK activity in these cells.

Previous reports demonstrated that a ratio of pro-apoptotic to anti-apoptotic proteins in PSCs is closer to the apoptotic threshold than the ratio in differentiated cells. Madden et al. found that differentiated cells express higher levels of the anti-apoptotic proteins, such as Bcl-2, than undifferentiated hESCs^61^. In contrast, undifferentiated hESCs were reported to express higher levels of pro-apoptotic proteins, such as Puma, than differentiated cells^62^. We have also found that the expression of anti-apoptotic factors Bcl-2 and Xiap is higher while the expression of the apoptotic executioner Caspase-3 is lower in miPSC-CMs than in undifferentiated PSCs (our unpublished data generated by Manoj K. Gupta). These data and cited studies indicate that murine and human PSCs are similarly more “primed” for apoptosis than differentiated cells. Lee et al. showed that perturbing the balance between pro- and anti-apoptotic proteins in hPSCs by inhibiting the anti-apoptotic factor survivin leads to apoptosis in a p53-dependent manner more readily in hPSCs than their differentiated derivatives^39^. Interestingly, Gao and coworkers reported that salicylic diamines down-regulate the expression of anti-apoptotic *Bcl-xL* and *Bcl-2* mRNAs in cancer cells^44^, suggesting that they might also affect the balance between pro- and anti-apoptotic proteins more readily in PSCs than in CMs, and in this way lead to selective killing of PSCs. However, we found no evidence that the toxic effect of SM6 in miPSCs is mediated by p53.

CM survival is predominantly regulated by signaling via Akt and Pim-1 kinases preventing mitochondria-mediated apoptosis in these cells^63^. Akt phosphorylates hexokinase-II which protects from mitochondrial permeability transition pore opening^64^, and Pim-1 attenuates calcium-induced mitochondrial swelling and cytochrome c release in CMs^65^. Moreover, Akt and Pim-1 are responsible for the inactivation of the pro-apoptotic Bcl-2 family member Bad^66^. In CMs Pim-1 enhances the expression of Bcl-xL and Bcl-2^65^, which may increase the resistance of PSC-CMs against salicylic diamines by compensating salicylic diamine-induced Bcl-xL and Bcl-2 down-regulation^44^. Therefore, differences in the activity of apoptotic and cell survival pathways as well as metabolic differences between PSCs and CMs most likely underlie the selective SM toxicity in PSCs.

In our experiments, neither SM6 nor puromycin completely eliminated PSCs in CMs that were used for transplantation. SM6 left only about 12 miPSCs in one million CMs while on average about 3 iPSCs were left in one million CMs after selection with puromycin. Since there was only a small difference in the number of contaminating PSCs in both of these CM preparations, it was surprising to find that none of 8 mice transplanted with puromycin-treated CMs developed teratoma even after 133 days of monitoring, while teratomas developed after 4 weeks of transplantation in five out of seven mice transplanted with SM6-treated CMs. These findings suggest that the threshold for teratoma formation in same species is very low and that only little differences in the number of contaminating PSCs in the CM population may determine whether the tumor will be formed or not. Partial inhibition of teratoma formation from miPSCs by SM6 in a mouse model indicates that it should be possible to further develop this already highly active compound for safe application in the clinic.

In order to achieve complete elimination of hiPSCs and simultaneously minimize cytotoxic effects on differentiated cells, fine-tuned optimizations of the small molecule concentration and the duration of application will be needed. Additionally, a combination of different PSC eliminating compounds or strategies may increase the effectiveness of PSC ablation in differentiated cell populations. Closer insights into the molecular mechanism of action of salicylic diamines will provide the experimental basis for the synthesis of SM derivatives that may possess even higher biological activity and selectivity. Furthermore, the selectivity of salicylic diamines for hPSCs might be increased by selecting the more appropriate developmental time window for treatment. It is known that during the differentiation process CMs transit from an initial immature state in which CMs primarily utilize glucose and the citric acid cycle in their metabolism to a more mature state at later stages of differentiation in which CMs primarily utilize fatty acid oxidation^67,68^. Since our findings suggest that SM6 is more toxic to cells that rely more on OXPHOS (miPSCs) than on glycolysis (hiPSCs) for energy production, it is likely that this and similar compounds will exert different levels of toxicity to immature and mature PSC-CMs, which needs to be explored further. In these ways, it might be possible to further optimize the use of salicylic diamines for purging hPSCs from CM preparations and completely eliminate the tumorigenic risk in clinical applications.

## Experimental procedures

### PSC culture and differentiation

The transgenic miPSC line αPIG-AT25 and mESC line αPIG44-D3 were described previously^48,69^. They express PAC and EGFP under the control of α-MHC promoter and allow for generation of highly pure EGFP-expressing CMs by puromycin treatment. To demonstrate the broad validity of the results the cytotoxicity experiments were also performed with the wild type mESC line R1 and hiPSC line NP0014-6 (UKKi007-A) which was described by us previously^70^. The names and characteristics of hiPSC lines used in this study are summarized in the Supplementary Table S1. Detailed cell culture and cardiac differentiation protocols for murine and human iPSC lines are available online in Supplementary Experimental Procedures.

### Cytotoxicity assays

mPSCs were seeded on 0.1% gelatin-coated 96-well plates at a density of 5000 cells/well and cultured in the complete mESC medium. After 1 day, cells were incubated with the indicated concentrations of various compounds as indicated in the respective figures and figure legends. The syntheses of the small molecules used in this study were described in previous publications as detailed in Supplemental Experimental Procedures. For treatment of CMs with SMs, puromycin-selected miPSC-CMs from day 14 of differentiation were dissociated into single cells as described above and plated on 5 µg/ml fibronectin-coated (PromoCell) 96-well plates at the density of 5 × 10^4^ cells/well. Human iPSC-CM monolayers were dissociated at day 43 of differentiation with Trypsin/EDTA and plated at 7.5–10 × 10^4^ cells per well of a Matrigel-coated 96-well plate. CMs were then cultured for an additional 2–3 days to form confluent spontaneously contracting monolayers and were then treated with indicated concentrations of SMs for 48 or 72 h in their respective medium. Following the treatment with SMs, the cell viability was determined with PrestoBlue cell viability reagent according to manufacturer’s recommendations (Life Technologies). Fluorescence intensities were measured in a GeniosPro microplate reader (Tecan) and the IC_50_ values were determined by regression analysis of dose–response curves upon fitting with the Boltzmann sigmoid function. Cell viability was also assessed microscopically using an Axiovert 100 inverted microscope and an Axiovert 200 M fluorescence microscope (both Carl Zeiss).

### Assessment of side-effects of SMs on PSC-CMs

Methods used for assessment of side-effects of SMs on purified PSC-derived CMs are described in detail in Supplementary Experimental Procedures. They include the methods for determination of CM beating frequency, apoptosis rate, DNA damage, reactive oxygen species (ROS) levels, subcellular localization of p53 and action potential properties.

### Oxygen consumption rate (OCRs)

The effect of SMs on mitochondrial respiration was determined by measuring OCRs with an XFe-96 extracellular flux analyzer (Seahorse Biosciences). miPSCs were plated on a gelatin-coated XFe96-well plate (Seahorse Bioscience) at a density of 3000 cells/well and cultured in mESC medium. Analysis of CMs was performed with purified day 16 miPSC-CMs plated on a fibronectin-coated XFe96-well plate (3 × 10^4^ cells/well) in differentiation medium. Cells were cultured for 2 days before the treatment to ensure adherence to the cell culture plate and then exposed to different concentrations of SMs for 16 h (miPSCs), 24 h (hiPSCs) or 48 h (miPSC-CMs). After washing with PBS, Seahorse assay medium was added to the cells and OCR was measured during three **c**ycles of measurement in 7 replicates per group using the Seahorse XF Cell Mito Stress Kit (Seahorse Bioscience). Following the measurement of basal respiration, the selective inhibitors oligomycin (1 µM), carbonyl cyanide-4-(trifluoromethoxy)-phenylhydrazone (FCCP, 1 µM) and antimycin A together with rotenone (1 µM each) were injected stepwise to quantify ATP production, maximal cellular respiration and the amount of non-mitochondrial respiration, respectively. Resulting OCR values were normalized to the relative number of cells in each well which was determined using PrestoBlue reagent.

### Assessing the mPSC contamination level by colony formation assay

To assess the potential of SM6 for complete elimination of remaining PSCs in mPSC-CMs, cardiac clusters were prepared by treatment of transgenic αPIG-mPSCs from day 9–14 of differentiation with 2–4 µg/ml of puromycin which was high enough to yield relatively pure CM populations but still low enough to permit a certain low level of contamination with undifferentiated PSCs. The ability of SM6 to eliminate these residual PSCs was then assessed by treating pre-purified day 14 cardiac clusters with 1, 5 or 10 M of SM6 for 2 days in the absence of puromycin. After treatment, cardiac clusters were enzymatically dissociated into single cells and the level of PSC contamination determined by plating 2 × 10^5^ cells on a layer of MEFs in a 6 cm cell culture dish. Cells were then cultured in ESC medium supplemented with LIF for 7–10 days and PSC colonies that emerged were visualized by staining with 1% crystal violet (Fluka Analytical, St. Louis, USA) in methanol (Applichem), and quantified.

### In vivo bioluminescence imaging of teratoma formation

In vivo studies were performed using the αPIG-AT25-derived miPSC line constitutively expressing firefly luciferase^50^. Animal experiments described in this study were approved by the Landesamt für Natur, Umwelt und Verbraucherschutz NRW (LANUV, Permit Number: 9.93.2.10.31.07.010 and 84-02.04.2013.A067), and conformed to the Directive 2010/63/EU of the European Parliament. Cells were differentiated as described above and CMs were selected with 2 µg/ml puromycin from day 9 until day 14 of differentiation followed by treatment with 10 µM SM6 for 2 additional days. Cardiac clusters were enzymatically dissociated and 1 × 10^6^ CMs were transplanted in 50 µl of PBS into the right hind limb muscle of male immunodeficient NSG mice. CMs isolated from untreated cardiac clusters and from clusters that were purified by using genetic selection with 8 µg/ml puromycin for seven days were transplanted as controls. The cell fate in all three groups was monitored by bioluminescence imaging in an IVIS200 system (Xenogen) as described by us before^50^. BL signal was quantified in the region of interest using Living Image 4.5 software (Perkin Elmer).

### Statistics

Statistical analyses were performed with Microsoft Excel software. P values for evaluation of significance between two groups were calculated via two-tailed paired Student’s t-test. P-values lower than 0.05 were considered significant and are shown in the figures as *p < 0.05, **p < 0.01, and ***p < 0.001. IC_50_ uncertainties (standard errors of the estimate, SEE) were calculated using standard errors of regression derived from logarithm-transformed regression analyses of dose response data. Data ratios are stated with propagated uncertainties calculated using standard deviations.

## Supplementary Information

Supplementary Information.

## Data Availability

All data generated or analyzed during this study are included in this published article (and its Supplementary Information files). All materials, data and associated protocols will be made available to any interested scientists.
