# Supplementary material for: Salicylic diamines selectively eliminate residual undifferentiated cells from pluripotent stem cell-derived cardiomyocyte preparations
Source: Sci Rep. 2021 Jan 27;11:2391. doi: 10.1038/s41598-021-81351-z (PMC7841182; doi:10.1038/s41598-021-81351-z)
Supplement: Supplementary file 1 — Supplementary Information. [file 41598_2021_81351_MOESM1_ESM.pdf]

Supplementary Information

Salicylic Diamines Selectively Eliminate Residual Undifferentiated Cells from Pluripotent Stem Cell-Derived Cardiomyocyte Preparations

Karsten Burkert, Hadiseh Taheri, Sarkawt Hamad, Matteo Oliverio, Gabriel Peinkofer, Jan-Wilhelm Kornfeld, Wacharee Harnying, Kurt Pfannkuche, Jürgen Hescheler, Albrecht Berkessel, and Tomo Šarić

Index

Supplementary Results	page 1
Supplementary Methods	page 6
Figure S1	page 18
Figure S2	page 19
Figure S3	page 20
Figure S4	page 21
Figure S5	page 23
Figure S6	page 25
Figure S7	page 26
Figure S8	page 28
Figure S9	page 30
Figure S10	page 31
Table S1	page 32
Table S2	page 33
Table S3	page 34
Table S4	page 35
Supplementary References	page 36

Supplementary Results

PluriSIn #1 is not highly toxic to murine PSCs

In order to determine the ability of the previously published PSC-eliminating compound PluriSIn #1 (Ben-David et al., 2013) to remove residual contaminating cells in murine PSC-CM preparations we treated murine αPIG-AT25 iPSCs, αPIG44-D3 ESCs and R1 ESCs with 40 µM PluriSIn #1 purchased from Sigma-Aldrich (catalog number SML0682). To verify the activity of the commercial compound, we have also synthesized fresh batches of PluriSIn #1 as well as fresh batches of eight chemical PluriSIn #1 derivatives (**Supplementary Fig. S1a**). While 8 µg/ml puromycin reduced the viability of mPSCs by 96.4±2.8% ($p<0.001$), the commercially available PluriSIn #1 and the in-house synthesized PluriSIn #1 reduced their viability by only 51.3±10.9% and 32.4±11.7%, respectively ($p<0.001$; **Supplementary Fig. S1b**). The derivatives of PluriSIn #1 showed inhibitory effects on the viability of PSCs comparable to the synthesized original molecule (average relative viability compared to DMSO control: 0.74±0.12 (P1), 0.74±0.19 (P3), 0.67±0.16 (P4); **Supplementary Fig. S1b**) or were not eliminating PSCs at all (average relative viability compared to DMSO control: 1.13±0.17 (P2), 0.95±0.39 (P5), 1.03±0.30 (P6), 0.85±0.16 (P7), 1.36±0.14 (P8); **Supplementary Fig. S1b**). PSCs were still detected by microscopic analysis after 3 days of treatment with 40 µM of PluriSIn #1 (**Supplementary Fig. S1c**) which is the concentration two-times higher than recommended for human PSCs.

The elimination of remaining PSCs in a PSC-derived cell population is a major obstacle for the clinical translation of stem cell research. We applied the well investigated PSC-eliminating small molecule for CM purification PluriSIn #1 (Ben-David et al., 2013; Lee et al., 2013) and various derivatives of it on murine ESCs and iPSCs. Even with double the concentration (40 µM) than recommended, none of the compounds was able to eliminate

murine PSCs after 72 h of treatment. It has been indicated before that PluriSIn #1 has decreased cytotoxicity on murine PSCs compared to human PSCs (Ben-David et al., 2013). Since PluriSIn #1 was identified to inhibit SCD1 in human PSCs (Ben-David et al., 2013) a differential regulation of SCD expression in mouse may be responsible for those variations. Indeed, four isoforms of SCD have been identified in mouse (SCD1, SCD2, SCD3, SCD4) while only two have been characterized in human (SCD1, SCD5) (Paton and Ntambi, 2009), but it is not known whether functional differences exist between these isoforms. It is also worth noting that the selective cytotoxicity of PluriSIn #1 was not consistent with human PSCs as well since Kropp and coworkers were not able to reproduce the PSC eliminating effect in experiments with confluent hPSC monolayers which they observed with five different cell lines (Kropp et al., 2015). Varying cell culture conditions like medium composition and involvement of feeder cells seem to repress the potency of PluriSIn #1 (Ben-David and Benvenisty, 2014; Ben-David et al., 2013) which make the achievement of best possible effects challenging.

SM structure-function relationship

Dose-response analyses performed with a newly synthesized batch of SM2, SM6 and SM8 corroborated initial findings and validated their toxicity towards miPSCs (**Supplementary Supplementary Fig. S3a**). Since SM6 possessed the lowest IC_{50} value for αPIG-AT25 miPSCs, additional dose-response analyses were performed with SM6 stereoisomers (**Supplementary Fig. S3b**) and ten SM6-based chemical structures C1-C10 (**Supplementary Fig. S3c**) in order to elucidate the structure-function relationship. Comparison of SM6 stereoisomers revealed slightly but not significantly higher toxicity of the initially tested R,R isomer compared to the S,S or the S,R isomers indicating that its activity is not affected by its spatial orientation (**Supplementary Fig. S3b**). Moreover, none of the ten tested SM6-based chemical structures exerted a significant toxicity against miPSCs (**Supplementary Fig. S3c**),

suggesting that the intact SM6 molecule and not its metabolic derivatives that could be formed within cells is required for its PSC-toxic activity.

Effect of SM6 on action potential (AP) properties of purified cardiac clusters

Electrophysiological analysis revealed same beating frequencies of SM6-treated and non-treated clusters ($p>0.05$; **Supplementary Fig. S6a**). Furthermore, action potential (AP) amplitude and maximal diastolic potential (MDP) were similar to control clusters as well ($p>0.05$; **Supplementary Fig. S6b,c**). Significant differences were observed in the AP durations (APDs) which increased when the clusters were treated with 5 or 10 µM of SM6 (**Supplementary Fig. S6d**). However, the ratios of APD50/APD90 were not significantly compromised (**Supplementary Fig. S6e**). Maximal upstroke velocities (Vmax) of APs slightly but not significantly decreased when clusters were treated with 5 and 10 µM of SM6 compared to non-treated clusters (**Supplementary Fig. S6f**). Taken together, overlays of APs derived from SM6-treated and non-treated cardiac clusters illustrate that SM6 had only minor electrophysiological effects on APs after 48 h of treatment (**Supplementary Fig. S6g**).

Elimination of PSCs in murine ESC-derived cardiac clusters with SM6

The morphology of murine ESC-derived cardiac clusters after SM6 treatment was similar to those of DMSO or puromycin-treated controls (**Supplementary Fig. S7b**). Interestingly, even though pre-purification was performed with double the concentration of puromycin (4 µg/ml) between day 9 and day 14 compared to that used in experiments with the αPIG-AT25 miPSCs (compare with the scheme in **Fig. 5a**), the number of residual PSCs in the DMSO control group on day 16 was 61.6±24.4-fold higher in ESC-derived clusters than in iPSC-derived clusters (compare **Fig. 5c,e** and **Supplementary Fig. S7c,d**) demonstrating an enhanced capacity of PSCs from this ESC line to survive in cardiac clusters in the presence of puromycin. Nonetheless, 48 h treatment with 5 and 10 µM of SM6 considerably reduced the

number of ESC-derived colonies by 93.8±3.5% (5 µM SM6) and 96.2±0.8% (10 µM SM6) compared to DMSO control, whereas the effect of 8 µg/ml puromycin and 1 µM SM6 on colony forming PSCs was minor and variable (47.8±24.9% reduction was achieved with puromycin and 20.0±26.9% with 1 µM SM6 compared to DMSO control, **Supplementary Fig. S7c,d**).

Comparing the relative cell yield in ESC-derived day 16 clusters, the number of cells generated in all treated groups was lower than in the DMSO control group (**Supplementary Fig. S7e**). However, the cell yield in the 8 µg/ml puromycin-treated group was lower (55.9±7.3% of the DMSO control) than in SM6-treated clusters (on average 72.9±8.9% of the DMSO group). As determined by flow cytometric analysis, the loss of cells in the drug treated clusters was due to a decrease in the $GFP^-/cTnT^-$ population of non-CMs. While 38% of cells in DMSO control group were non-CMs this fraction was reduced to 12% in puromycin group and to 31%, 12% and 8% in 1 µM, 5 µM and 10 µM SM6-treated groups, respectively (**Supplementary Fig. S7f**). Interestingly, a population of $GFP^-/cTnT^+$ CMs in ESC-derived clusters was 1.7-fold larger in SM6-treated than in puromycin-treated clusters (**Supplementary Fig. S7f**). Plating ESC-derived CMs after SM6 treatment demonstrated the capability of these cells to form α-actinin expressing CM monolayers (**Supplementary Fig. S7g**) and staining for the PSC marker Oct4 showed the presence of pluripotent cells after treatment with 1 µM and 5 µM of SM6 but not after treatment with 10 µM of SM6 (**Supplementary Fig. S7h**).

Taken together, by using SM6 in large-scale suspension cultures of mESCs (**Supplementary Fig. S7)** and miPSCs (**main Figures 5 and 6**), we demonstrated that it was possible to reduce the number of contaminating PSCs in murine ESC- and iPSC-derived cardiac cell aggregates by more than 96-99%. This efficiency was comparable to that of the antibiotic-based genetic selection method and did not compromise the CM viability or their functional properties.

Furthermore, even when used alone, SM6 was capable of eliminating PSCs in differentiating EB cultures to the same extent as the puromycin-based genetic selection method. Moreover, the yield of CMs in SM6-treated EB cultures was higher than that obtained with puromycin because SM6, but not puromycin, preserved the subpopulation of cTnT-positive CMs in which the transgenic αMHC-promoter-driven selection markers were not expressed. This puromycin-sensitive and GFP-negative CM subpopulation most likely represents ventricular CMs because the αMHC promoter in the developing rodent heart is predominantly active in the atrium whereas in ventricles this isoform is activated only after birth (England et al., 2013). This is also in agreement with Kolossov et al. who found that the αMHC promoter in mESC-CMs was active only in pacemaker and atrial but not in ventricular CMs (Kolossov et al., 2006). Thus, in murine system, the small molecule based elimination of PSCs has significant advantage over genetic selection because it preserves more heterogeneous CM population independently of subtype and results in higher CM yields than the genetic approach.

Supplementary Methods

Cell culture

The transgenic miPSC line αPIG-AT25 and mESC line αPIG44-D3 were cultured on mitomycin-inactivated mouse embryonic fibroblasts (MEF) in maintenance medium composed of Dulbecco's minimal essential medium (DMEM) with GlutaMAX (Life Technologies, Carlsbad, USA) supplemented with 15% fetal bovine serum (FBS, Sigma-Aldrich, St. Louis, USA), 1× non-essential amino acids (NEEA, Life Technologies), 50 µM β-mercaptoethanol (β-ME, Life Technologies) and 1000 U/ml of murine leukemia inhibitory factor (LIF, ORF Genetics, Kopavogur, Iceland). Cell passaging was performed every 2-3 days by dissociating the cells with 0.05% trypsin-EDTA (Life Technologies) and seeding 1-2×10^4 cells/cm^2. The wild type mESC line R1 was cultured on cell culture plates coated with 0.1% gelatin (Sigma-Aldrich) and maintained in the standard mESC medium like the transgenic mPSCs.

Human iPSCs lines NP0014-6 (UKKi007-A), NP0040-8 (UKKi011-A) and NP0141-31B (UKKi032-C) used in this study were generated by us using insertional (NP0014-6 iPSC line) or non-insertional (NP0040-8 and NP0141-31B iPSC lines) reprogramming technologies as detailed in **Table S1** (Fatima A. et al., 2011; Zhang X. H. et al., 2013; Zhang X. H. et al., 2019; Hamad S. et al., 2019). These lines are deposited at the European Bank for hiPSCs (EBiSC, https://ebisc.org) and are registered at the online hiPSC repository hPSC^{reg} (https://hpscreg.eu/). All three hiPSC lines were maintained in E8 medium (Life Technologies) on cell culture plates pre-coated with 5 µg/ml vitronectin (Life Technologies). At 70-80% of confluence, hiPSCs were passaged as small aggregates every 4-5 days at 1:20 splitting ratio after dissociation with 0.48 mM Versene solution (Life Technologies). Medium was changed every day.

Cardiac differentiation of murine iPSCs and ESCs

The differentiation of transgenic mPSCs was initiated in mass cultures by inoculating one million PSCs into 14 ml of differentiation medium composed of Iscove's Modified Dulbecco's Medium (IMDM) with GlutaMAX, 20% fetal bovine serum (FBS), 1× non-essential amino acids (NEAA), 100 µM β-mercaptoethanol (β-ME) (all from Life Technologies) and 50 µg/ml L-ascorbic acid phosphate (Wako) in 10 cm non-adherent plate. Cells were cultured on a horizontal shaker and on day 2 of differentiation, embryoid bodies (EBs) were transferred into a 250 ml spinner flask (CELLSPIN 250, Integra Biosciences) at a density of 3×10^4 EBs per 200 ml of differentiation medium lacking ascorbic acid. On day 9 of differentiation, purification of CMs was initiated by adding puromycin (Life Technologies) to final concentration of 8 µg/ml. Two days later, CM clusters were transferred into non-adherent 10 cm dishes containing fresh differentiation medium and 8 µg/ml puromycin. If not otherwise specified, CM clusters were further cultured in the presence of puromycin on a shaker until day 16 of differentiation with medium change every 2 days. For single cell analyses and establishing monolayer CM cultures, clusters were dissociated with 0.25% trypsin-EDTA (Life Technologies) and 50 U/ml DNase I (AppliChem) for 20-30 min at 37°C. The CM suspensions were filtered through a membrane with 60 µM pore size (Sefar) to remove larger cell aggregates, cells were counted and stored on ice until further use. The purified Cor.4U hiPSC-derived CMs (hiPSC-CMs) were kindly provided by Axiogenesis (Cologne, Germany) and cultured following the manufacturer's recommendations.

Cardiac differentiation of human iPSCs

Prior to cardiac differentiation the NP0040-8 hiPSC line was cultured in E8 medium in 6-well plates coated with 10 µg/cm^2 Matrigel Matrix (hESC-qualified, Corning, Cat. # 734-1440) in a humidified incubator at 37°C and 5% CO_2. The E8 medium was supplemented with 5 µM of

Rho Kinase (ROCK) inhibitor Y27632 (AdooQ Bioscience, Cat. # A11001-5) for the first two days after each passage. Once the hiPSC-confluency had reached about 80%, the medium was discarded, cells washed with Dulbecco's phosphate buffered saline without calcium and magnesium ($DPBS^{-/-}$) and hiPSC colonies were dissociated into single cells by adding 1 mL of 0.48 mM Versene into each well of a 6-well plate and incubating at 37°C for 7 minutes. After counting, 70,000 cells/cm^2 were seeded onto Matrigel-coated 6-well plates and maintained in 2 mL of E8 medium supplemented with 5 µM ROCK inhibitor for two days. After two days, fresh E8 medium without ROCK inhibitor was exchanged and cells were cultured for additional two days. The culture medium was then changed to RPMI 1640 containing 1× B27 supplement without insulin (Thermo Fisher Scientific, Cat. # A18956-01), 50 µg/mL L-ascorbic acid phosphate magnesium n-hydrate (Wako Chemicals Europe, Cat. # 013-12061) and 8 µM CHIR99021 (LC Laboratories, Cat. # C-6556). This day was defined as day 0 of differentiation. After exactly 24 hours, the medium was completely changed to RPMI 1640 containing 50 µg/mL ascorbate and 1× B27 supplement without insulin. At 72 hours after initiation of differentiation half of the medium was changed to RPMI 1640 supplemented with 1× B27 without insulin, 50 µg / mL ascorbate, 5 µM IWP2 (Tocris, Cat. # 3533 / 10) and 5 µM XAV939 (Sigma-Aldrich, Cat. # X3004) and the cells were further cultured for 48 hours. From day five onwards the culture medium was changed to RPMI 1640 containing 1× B27 without insulin and 50 µg/mL ascorbate. Spontaneously beating cardiomyocytes appeared on days 7-10. From then on they were maintained in the CM-maintenance medium composed of RPMI 1640 supplemented with 1× B27 without insulin, 50 µg/mL ascorbate and 1× penicillin and streptomycin (Gibco, Cat. # 15140-122). Complete medium change was performed every 2-4 days.

Dissociation of hiPSC-CMs

At the specified day of differentiation, the culture medium was washed with DBPS$^{-/-}$ and CMs were dissociated by using 0.05% Trypsin-EDTA (Thermo Fisher Scientific, Cat. # 25300-054) for 25 minutes at 37°C. The Trypsin was inhibited with 0.5% bovine serum albumin in DMEM:F12 and the dissociated cells were passed through an EASYstrainer filter with 40 µm pore size. Cell number was determined in an automatic cell counter and CMs were used for downstream analyses.

Small molecule syntheses

The salicylic diamines (salans) SM1, SM3, SM4, SM6, SM8 and SM9 were prepared as described by Butsch et al. (Butsch et al., 2013). The salalens SM2 and SM5 were prepared as described by Berkessel et al. (Berkessel et al., 2007). The compounds SM1, SM4 and SM8 and the compounds SM2 and SM5 represent stereoisomers of the same molecular formula. The bis-salicylic amide SM7 was prepared as described by Jiménez et al. (Jiménez and Belmar, 2005). Squaric acid derivative SM11 was prepared according to Zhu et al. (Zhu et al., 2010), whereas SM12-16 were prepared according to Eröksüz et al. (Eröksüz et al., 2017).

Compounds C1 and C10 are commercially available bulk chemicals. The tetramethyldiamines C2 and C3 were prepared according to Cabello et al. (Cabello et al., 2005), while the N,N'-dibenzylated diamines C4-C7 were prepared according to Gajewy et al. (Gajewy et al., 2012). Procedures for the synthesis of compounds C8 and C9 can be found in the reference of Berkessel et al. (Berkessel et al., 2007).

The nicotinoyl and iso-nicotinoyl hydrazides PluriSIn #1, P1, P3, P5 and P7 were prepared by standard coupling of the corresponding hydrazines with acid chlorides in the presence of triethylamine. The same holds for the benzoyl hydrazine P4. The PluriSIn #1 derivatives P2, P6 and P8 were prepared by reductive alkylation of iso-nicotinoylhydrazide with

benzaldehyde, cyclohexanone and cycloheptanone, respectively, in the presence of sodium borohydride. Synthesized PluriSIn #1 and derivatives were applied on mPSCs and compared to PluriSIn #1 purchased from Sigma-Aldrich (catalogue number SML0682). For all compounds (PluriSIn#1, P1-8), the analytical and spectroscopic data were in agreement with the structures shown in **Supplementary Fig. S1a**, and confirmed the purity of the materials synthesized.

All substances were diluted in DMSO at the final stock concentration of 50 mM (PluriSIn #1 and its derivatives P1-8) or 20 mM (all 16 SM compounds SM1-SM16). For mPSC-cytotoxicity assays, serial dilutions of DMSO stock solutions were prepared with cell culture medium.

Immunocytochemistry (ICC)

For ICC analyses, miPSC-CMs were plated on fibronectin-coated multi-well plates (1×10^5 cells/cm^2) and treated with 8 µg/ml puromycin for two more days as described above. CM monolayers were then incubated with 1 or 10 µM of SMs for 48 h. CMs treated with bleomycin (20 µg/ml) for 3-5 h served as positive control for detection of DNA damage while CMs treated with cisplatin were used as positive control in analyses of structural integrity (150 µg/ml cisplatin for 48 h) and apoptosis (75 µg/ml cisplatin for 24 h).

The integrity of sarcomeric structures in murine and human iPSC-CMs was determined by α-actinin staining directly after 48 h of SM treatment. Detection of DNA damage in murine and human iPSC-CMs was done using γ-H2AX antibody and quantification of apoptosis rate was performed using cleaved caspase-3 antibody directly after 48 h of SM treatment. Murine iPSC-CMs were also analyzed after 72 h of recovery in culture medium without SMs. The pluripotency of undifferentiated mPSCs and hPSCs was confirmed by using the Oct4, Nanog, SSEA-4 and TRA-1-81 antibodies, respectively. Detailed information about primary and

secondary antibodies and their dilutions used in ICC studies is provided in the **Supplementary Table S4**.

For α-actinin staining, CMs were fixed with an ice-cold solution of methanol and acetone (1:1) for 10 min at -20°C and permeabilized with 0.2% Triton-X100 (Sigma-Aldrich) for 5 min. Cells were then blocked with 5% bovine serum albumin (BSA, Applichem) in PBS for 1 h at room temperature (RT) and incubated with the α-actinin antibody in 0.8% BSA in PBS overnight at 4°C. Cells were then washed with PBS and incubated in 0.8% BSA in PBS for 1 h at RT with the matching Alexa Fluor 555-conjugated secondary antibody containing Hoechst 33342 at 1:1000 dilution.

For γ-H2AX, cleaved caspase-3, Oct4, Nanog, SSEA-4 and TRA-1-81 stainings, cells were fixed with 4% paraformaldehyde (PFA, Morphisto) for 15 min at RT, permeabilized with 0.25% Triton-X100 and 0.5 M NH_4Cl (Roth) in PBS for 15 min at RT and blocked with 5% BSA in PBS for 1 h at RT. Incubations with primary antibodies were carried out in 0.8% BSA in PBS overnight at 4°C. Cells were washed with PBS and incubated in 0.8% BSA in PBS for 1 h at RT with the matching Alexa Fluor-conjugated secondary antibodies containing Hoechst 33342 at 1:1000 dilution.

After removing the secondary antibody and washing with PBS, cells were maintained in glycerol / PBS solution (1:1) containing 1% 1,4-Diazabicyclo[2.2.2]octan (DABCO, Sigma-Aldrich). Fluorescence images were captured with a Zeiss Axiovert 200M fluorescence microscope and analyzed with Zeiss Axiovision 4.5 software. For confocal laser scanning microscopy (LSM) cells were plated on µ-plates (Ibidi). Confocal images were taken with a Zeiss Meta 510 confocal laser scanning microscope (Carl Zeiss) using ZEN 2009 software (Carl Zeiss) and analyzed with Image J software (http://imagej.nih.gov/ij/).

Flow cytometry of murine cells

Determination of CM purity in the cell population was performed by flow cytometric analysis. PSC-derived EBs or cardiac clusters were dissociated with 0.25% trypsin-EDTA. Dead cells were stained with LIVE/DEAD fixable dead cell stain kit (Life Technologies) prior to cell fixation in 4% PFA for 15 min. Immunological staining of cardiac Troponin T (cTnT) was performed in order to detect the total CM population including GFP-negative CMs. To this end, 5×10^5 cells were permeabilized in PBS containing 1% saponin and 5% BSA for 1 h at room temperature. A cTnT mouse antibody (sc-20025, Santa Cruz, Dallas, USA) and the corresponding isotype control (sc-3878, Santa Cruz) were diluted 1:50 in the staining buffer (PBS containing 1% saponin and 0.8% BSA) and incubated for 30 min at 4°C. After washing the cells with PBS, cells were incubated with a goat anti-mouse IgG Alexa Fluor 555-conjugated secondary antibody (1:100, A21422, Life Technologies) in the same staining buffer for 1 h at 4°C. Pluripotent cells were stained by incubating 5×10^5 of non-fixed cells with an SSEA-1 antibody (sc-21702, Santa Cruz) or isotype control (sc-3881, Santa Cruz) diluted 1:20 in in PBS containing 1% FBS for 20 min at 4°C. After washing with PBS cells were incubated with the goat anti-mouse IgM Alexa Fluor 555-conjugated secondary antibody (1:100, A21426, Life Technologies) in PBS containing 1% FBS for 20 min at 4°C. Cells were measured in an Attune acoustic focusing cytometer (Life Technologies) and data was analyzed with the Attune Cytometric software v1.2.5 (Life Technologies). Marker expression was analyzed in a gated population of 10^4 viable cells.

Flow cytometric analysis of human iPSC-CMs

1.5×10^6 hiPSC-CMs were added into a 15 mL Falcon tube, washed with 1 mL DPBS$^{-/-}$ and centrifuged at 200×g for 4 minutes. The supernatant was discarded, the cells fixed in 1% paraformaldehyde (PFA) for 15 minutes, and centrifuged at 200×g for 4 minutes. hiPSC-CMs were re-suspended in 1 mL of 90% methanol (-20°C) and left for 20 minutes at 4°C for

further fixation and permeabilization. hiPSC-CMs were centrifuged at 200×g and washed by 2 mL DPBS$^{-/-}$ to remove residual methanol and then the pellet was incubated for one hour in 100 µL of blocking buffer (0.5% BSA and 0.1% Triton X-100 in DPBS$^{-/-}$) containing 1:50 dilution of anti-troponin T (TNNT2) mouse monoclonal IgG2a (Santa Cruz Biotechnology, Cat. # Sc-20025) or normal mouse monoclonal IgG2a antibody (Santa Cruz Biotechnology, Cat. # 3878). After washing in blocking buffer the cells were incubated for 30 minutes in 100 µL of blocking buffer containing 1 µg/mL secondary antibody Alexa Fluor 555 goat anti-mouse IgG (H+L) (Thermo Fisher Scientific, # A21422) at room temperature. hiPSC-CMs were washed two times with 2 mL of DPBS$^{-/-}$, the final cell pellet was re-suspended in 250 µL DPBS$^{-/-}$ and cells analyzed by flow cytometry in LSR Fortessa Analyzer (BD Biosciences). Analysis was performed using FCS express 6 (De Novo Software, Glendale, CA).

Whole cell lysate preparation

Murine iPSCs or iPSC-CMs were treated for 8 h with 10 µM SM6 or vehicle (0.05% DMSO). After treatment cells were collected, washed once with DPBS$^{+/+}$ and resuspended in 400 µl of ice-cold RIPA buffer (1% (v/v) NP-40, 5% sodium deoxicholate, 0.1% SDS, in PBS, pH 7.4). Cells were disrupted by pipetting with the yellow pipette tip 10 times. The homogenate was sonicated twice for 5 sec per cycle in order to completely disrupt the cells and to shred the DNA. Samples were incubated on ice for 15 minutes to generate a whole cell lysate (WCL). Control WCLs of human HEK293, HT29 and COS9 cells were prepared in the same way. Lysates were then centrifuged at 10,000 xg for 10 min at 4°C and the supernatant was collected. Protein concentration was determined by Pierce BCA Protein Assay kit (Thermo Fisher Scientific), aliquots were snap-frozen in liquid nitrogen and then stored at -80°C until use.

Subcellular fractionation

For fractionation into nuclear, mitochondrial and cytosolic fractions cells cultured in 6 cm plates were collected after 8 h of treatment with SM6- or 0.05% DMSO, washed with DPBS$^{+/+}$ and resuspended in 500 µl of ice-cold subcellular fractionation buffer containing 250 mM sucrose, 20 mM HEPES (pH 7.4), 10 mM KCl, 2 mM $MgCl_2$, 1 mM EDTA, 1 mM EGTA, 1 mM DTT and 1x Protease Inhibitor Cocktail, cOmplete Mini (Roche). Cells were disrupted by passing cell suspension ten times through a 27G needle using 1 mL syringe and lysates subsequently incubated on ice for 20 min. Samples were then centrifuged at 720 x *g* for 5 min at 4°C to collect nuclei in the pellet. The supernatant containing cytoplasm, membranes and mitochondria was transferred into a fresh tube on ice and centrifuged at 10,000 x *g* for 5 min at 4°C to pellet the mitochondrial fraction. The supernatant containing the cytoplasm and membrane fraction was aliquoted into a new tubes sitting on ice. The nuclear pellet from previous step was washed with 500 µl of subcellular fractionation buffer and passed through a 25G needle ten times. After centrifugation at 720 x *g* for 10 min at 4°C the supernatant was discarded and the pellet containing nuclei was resuspended in 300 µl of Tris-buffered saline (TBS) containing 0.1% SDS, sonicated briefly to shear genomic DNA and homogenize the lysate. The mitochondrial pellet was processed as described for the nuclear pellet to obtain mitochondrial lysate in TBS/0.1% SDS. Protein concentration in samples was determined using Pierce BCA Protein Assay kit (Thermo Fisher Scientific).

Immunoblotting

For immunoblotting, protein samples (14 µg/lane) and the SeeBlue Plus2 Pre-Stained Standard (Life Technologies) were loaded onto 12% separating polyacrylamide gel. After electrophoresis they were transferred onto PVDF membrane using the iBlot system (Life Technologies). After blocking for 60 min at room temperature in buffer containing 0.1 % Tween 20 and 0.5 % milk powder in TBS, pH 7.5, the membrane was incubated overnight at

4°C with primary antibodies against p53, cytosolic marker α-tubulin, or mitochondrial marker voltage-dependent anion channel (VDAC) (see **Table S4**). After washing with TBS, membrane was incubated for 1 h at room temperature with fluorescently conjugated secondary anti-rabbit IgG or anti-mouse IgG polyclonal antibodies in 0,5% milk in TBS (see **Table S4**). Membrane was then washed in TBS, dried and the near infrared (NIR) fluorescent signals were detected on Odyssey CLx Gel Imaging Scanner (LI-COR Biosciences) equipped with the Image Studio software. Full-length blots of all analyses are shown in **Supplementary Figs. S8, S9 and S10**.

Quantitative RT-PCR

mRNA was isolated from miPSCs or miPSC-CMs with TRIzol reagent (Thermo Fisher Scientific) and cDNA was synthesized from 1 µg mRNA using SuperScript First-Strand synthesis system (Life Technologies). The reaction was performed in 30 µl at 42°C for 50 min followed by the denaturation step for 10 min at 72°C. For quantitative PCR, 10 µl reactions were prepared in a 96-well plate containing 1× of SYBR Advantage qPCR Premix (Clontech, Mountain View, USA), 1:25 diluted cDNA and 0.2 µM of forward and reverse Oct4, Lin28 or GAPDH primers (Sigma-Aldrich). The PCR was performed in a 7500 Fast Real Time PCR System (Applied Biosystems, Carlsbad, USA) and data analyzed with SDS software v1.4.0 (Applied Biosystems). Initial denaturation at 95°C for 10 seconds was followed by 40 cycles at 95°C for 10 seconds and 60°C for 35 seconds. Relative expression of Oct4 and Lin28 genes was normalized to GAPDH gene expression.

Determination of the CM beating rate

The effect of SM treatment on CM beating frequency was determined by video imaging of iPSC-CM monolayers after exposure to SMs. The CMs were prepared and treated with SMs as described in the chapter Cytotoxicity Assays above. Following SM treatment, day 16

mPSC-CMs were washed twice with PBS and cultured for 72 h in fresh differentiation medium without SMs. Videos of CM monolayers were recorded immediately after 48 h of SM treatment and after 72 h of recovery using an Axiovert 100 microscope and a DFW-X710 digital camera (Sony). The number of beats per each video sequence was quantified and the contraction rate was calculated in beats per minute.

Apoptosis rate and DNA damage

Quantification of apoptosis rate and DNA damage was performed by immunocytochemistry (ICC) using, respectively, the antibody against cleaved caspase-3 and phosphorylated histone H2A variant H2A.X (γ-H2A.X), which is a surrogate marker of double-strand breaks. Cells were stained either directly after 48 h of SM treatment or after subsequent 72 h of recovery in culture medium without SMs. Detailed ICC protocol and information about antibodies used in ICC studies is provided in the **Supplementary Methods** and **Supplementary Table S4**.

Reactive oxygen species (ROS)

The ROS levels in miPSC-CMs after treatment with various compounds was determined by using CellROX Deep Red reagent (Life Technologies). Puromycin-selected miPSC-CMs from day 14 of differentiation were plated on a fibronectin-coated multiwell cell culture plate (10^5 cells/cm^2) and incubated with 8 µg/ml puromycin for 2 additional days to remove all non-CMs. CMs were then treated with SMs for 48 h or with 75 µg/ml cisplatin for 24 h, which served as positive control. The cells were then incubated with 5 µM of CellROX for 30 min at 37°C as recommended in the manufacturer's protocol. Nuclei were counterstained by 15 min incubation with Hoechst 33342. Cells were washed 3 times with PBS prior to determination of ROS-induced fluorescence in live cells by using a Zeiss Axiovert 200M fluorescence microscope. Mean fluorescence intensities of three images from areas containing

intact iPSC-CM monolayers were determined and compared to DMSO-treated control cells using Image J software.

Action potential (AP) recordings

APs were measured in spontaneously beating cardiac clusters from day 16 of differentiation using sharp glass microelectrodes with a resistance of 20-50 MΩ when filled with 3 M KCl (World Precision Instrument, Sarasota, USA). The intracellular recordings were performed as described before (Halbach et al., 2006) and signals were acquired with a SEC-10LX amplifier (npi electronic, Tamm, Germany) and Pulse software (HEKA, Lambrecht/Pfalz, Germany). Data was analyzed with Mini Analysis Program (Synaptosoft, Fort Lee, USA).

Teratoma histology

Mice were sacrificed and hind limbs were dissected, fixed with 4% PFA overnight at 4°C), immersed in 30% sucrose overnight at 4°C and cryopreserved in Tissue-Tek O.C.T. (Sakura Finetek), snap frozen in 2-methylpropane (Roth) placed in liquid nitrogen and stored at -80°C. Histological sections of 8 µm thickness were sliced with a cryotome CM-1950 (Leica) and stained with hematoxylin and eosin (Thermo Fisher Scientific).

Supplementary Figures

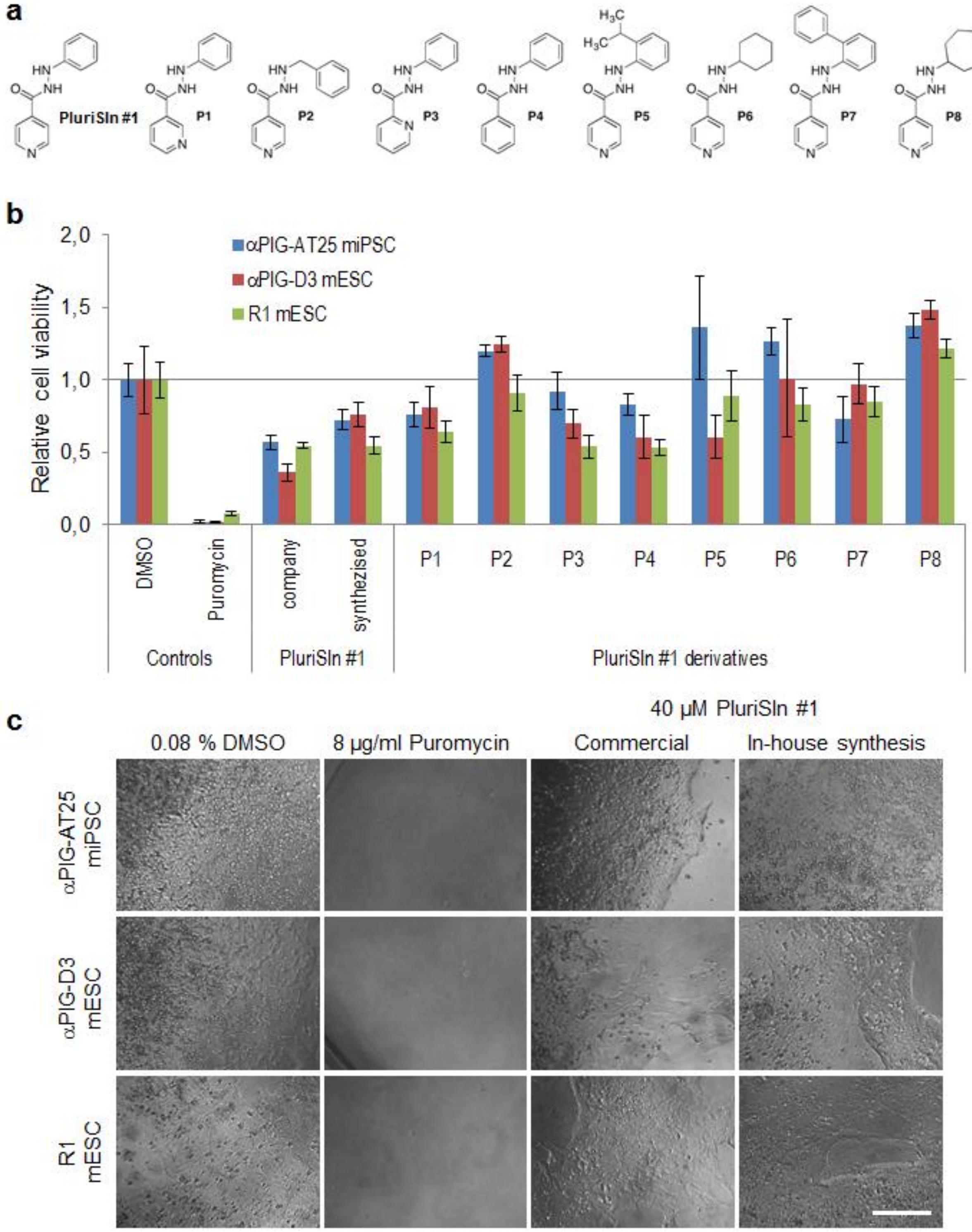

Figure S1. Murine PSCs treated with 40 µM of PuriSIn #1 and derivatives P1-P8 for 72 h. (a) Molecular structures of PluriSIn #1 and the derivatives P1-P8. (b) Viability of mPSCs after 72 h of treatment relative to 0.08% DMSO control (mean ± SD; n=4). Positive control cells were treated with 8 µg/ml puromycin. (c) Representative images of mPSCs after 72 h of treatment with 40 µM of a commercially available and an in-house synthesized PuriSIn #1. Scale bar: 50 µm.

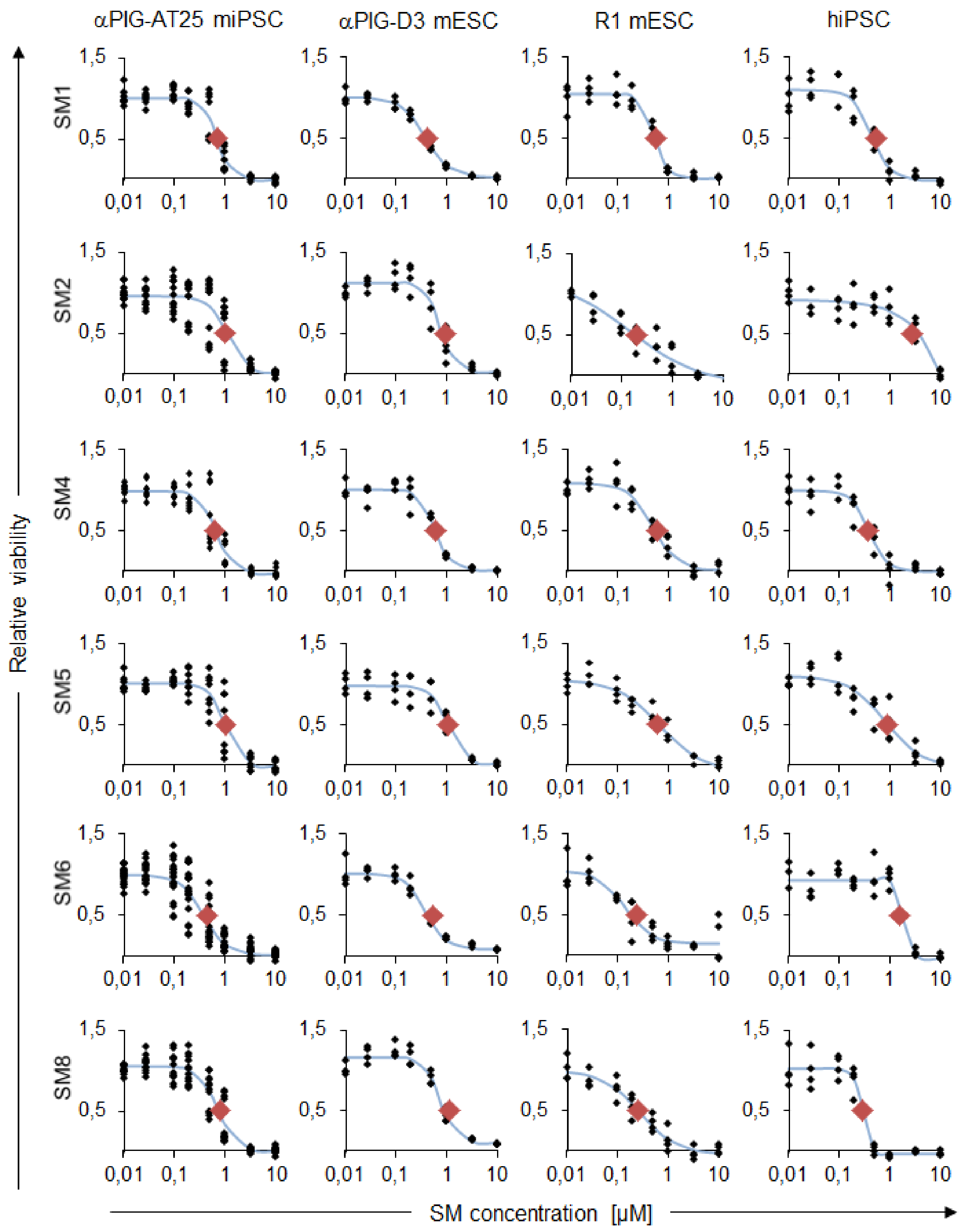

Figure S2. Dose-response curves for cytostatic effects of indicated SMs on three murine PSC lines (αPIG-AT25 iPSCs, αPIG-D3 ESCs, and R1 ESCs) and one human iPSC line (NP0014-6). Cell viability was determined after 48 h of treatment with 0.01, 0.03. 0.1, 0.2, 0.5, 1.0, 3.3 and 10.0 µM of each SM. Results are presented as relative values compared to untreated control cells. Data points represent individual measurements (n=4-20). Curve fitting was performed using the Boltzmann sigmoid function. The IC_{50} values are indicated by a red diamond. See also **Table S2** and **Table S3** for exact IC_{50} values for three human and three murine PSC lines, respectively.

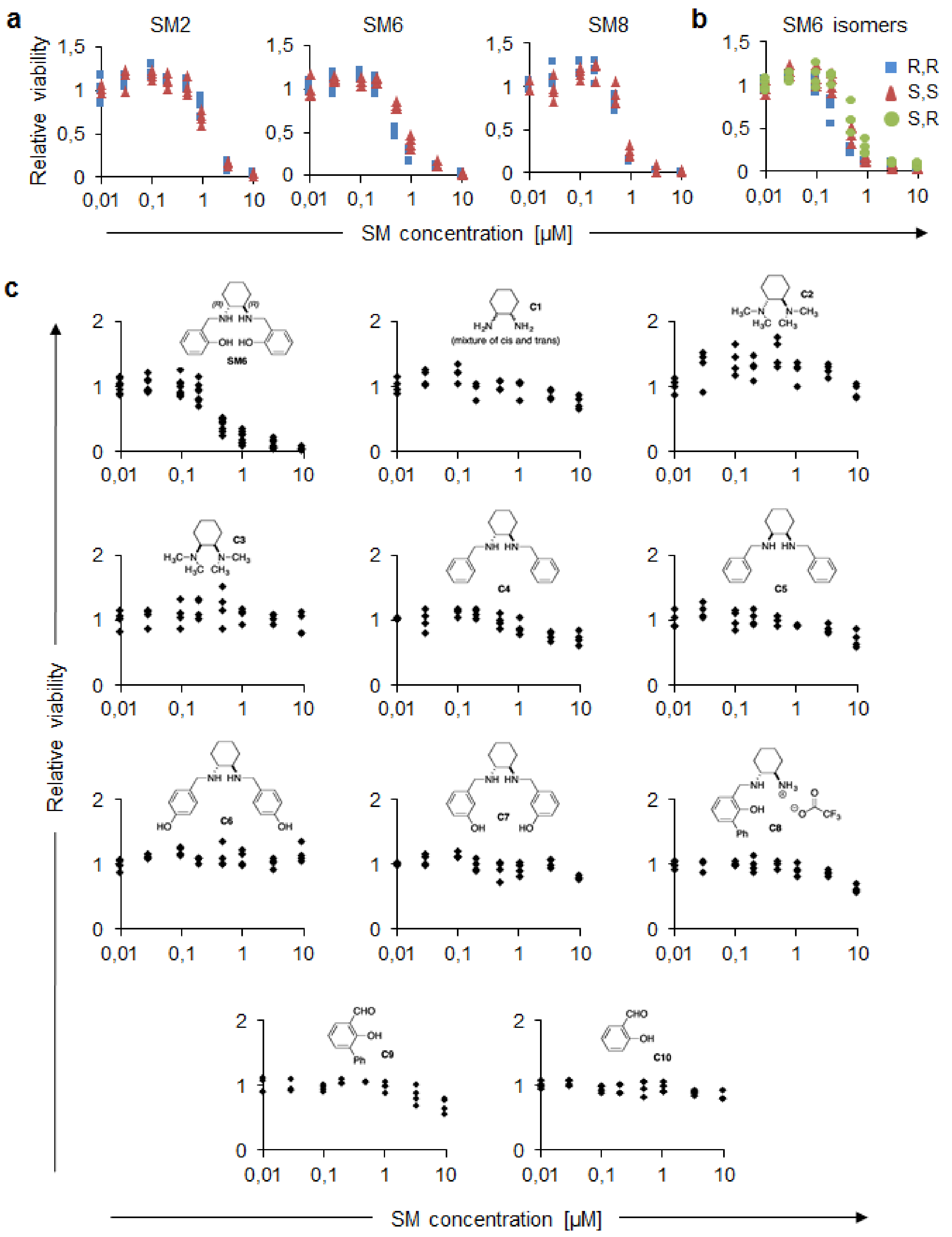

Figure S3. mPSC-cytotoxic effects of SM6 and its structurally related compounds and stereoisomers. (a) Relative viabilities of αPIG-AT25 miPSCs exposed for 48 h to SM2, SM6 and SM8 derived from two different batches of molecule synthesis and (b) to SM6 stereoisomers (n=4). Differentially colored symbols indicate different batches (in a) or different stereoisomers (in b). SM6 is in an R,R conformation. (c) Relative viabilities of αPIG-AT25 miPSCs exposed for 48 h to SM6 and ten structurally related compounds C1-C10 (n=4).

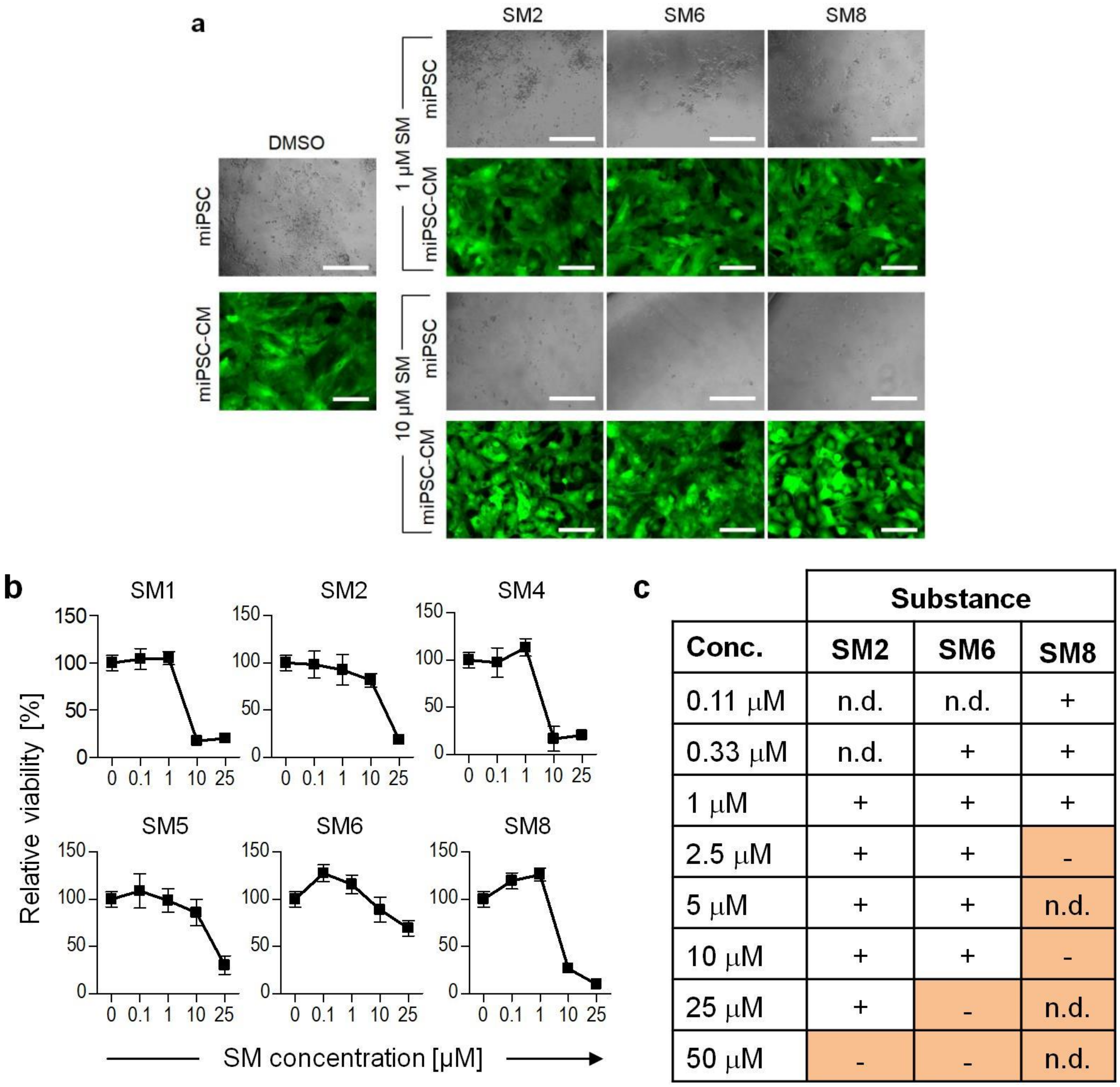

c

Conc.	Substance SM2	SM6	SM8
0.11 µM	n.d.	n.d.	+
0.33 µM	n.d.	+	+
1 µM	+	+	+
2.5 µM	+	+	-
5 µM	+	+	n.d.
10 µM	+	+	-
25 µM	+	-	n.d.
50 µM	-	-	n.d.

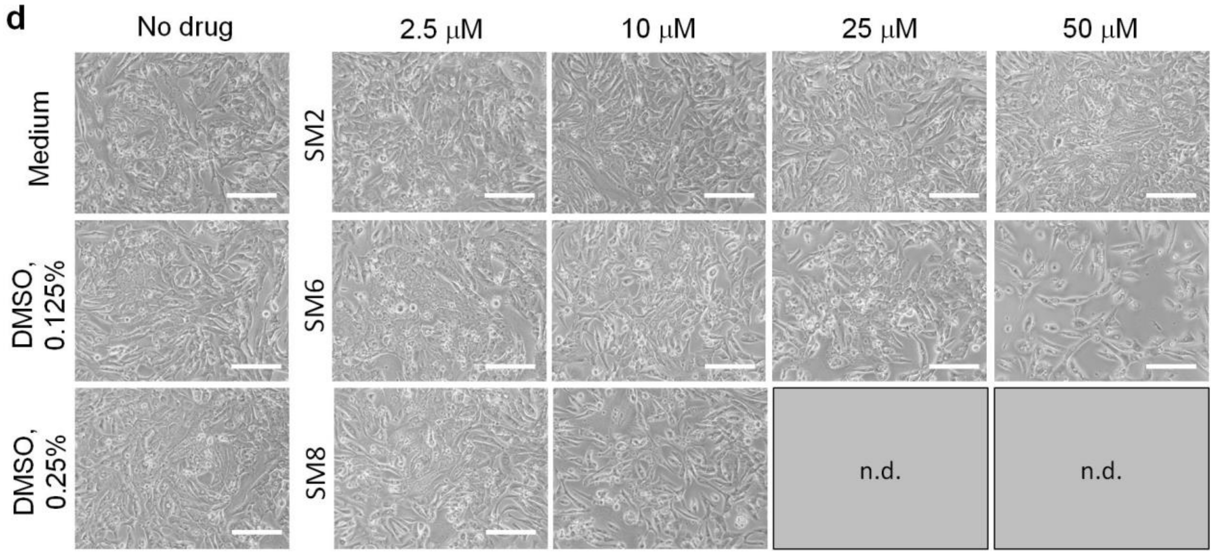

Figure S4. Cytotoxicity of indicated SMs against murine and human iPSCs and iPSC-CMs. (a) Microscopic images of αPIG-AT25 miPSCs (bright-field) and purified miPSC-CMs expressing GFP under control of cardiac α-MHC-promoter treated for 48 hr. Control cells were treated with 0.05% DMSO. (b) Viability of αPIG-AT25 miPSC-CMs treated with SMs

for 72 h relative to DMSO-treated control (mean ± SD; n=4). (c) Microscopic assessment of the spontaneous beating activity of plated NP0040 hiPSC-CMs (day 46 of differentiation) after 48 h of treatment with indicated SMs. n.d. – not determined. (d) Representative bright field images of NP0040 hiPSC-CMs (day 46 of differentiation) after 48 h treatment with indicated SMs or controls. Scale bars in a and d: 100 µm.

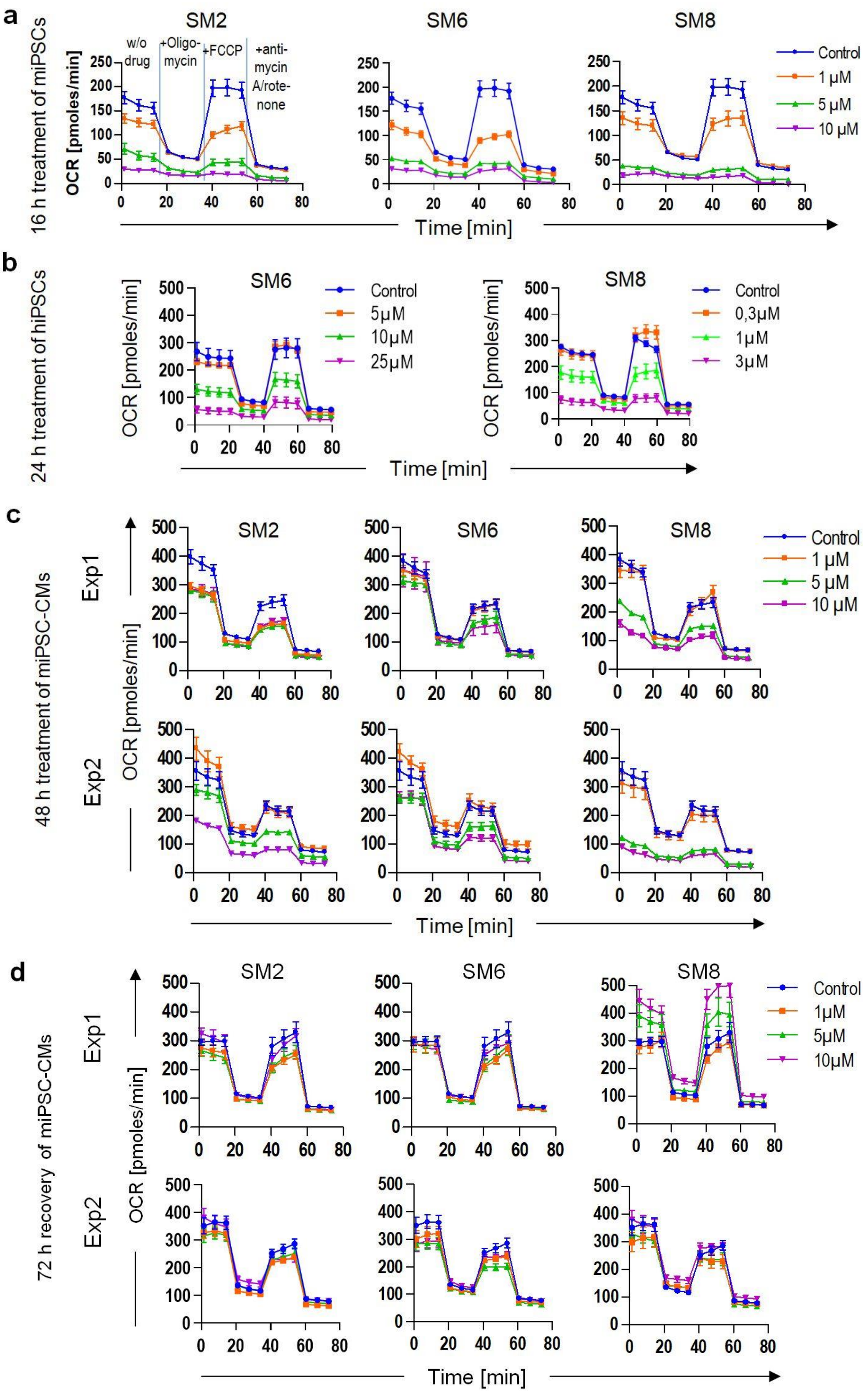

Figure S5. Oxygen consumption rates (OCRs) in miPSCs and purified miPSC-CMs as well as in hiPSCs after SM treatment. (a) OCRs after 16 h of SM treatment of αPIG-AT25 miPSCs (mean ± SEM; n=7). Control cells were treated with 0.05% DMSO. Measurement of

basal respiration (measuring points 1-3) was followed by injection of ATPase inhibtor oligomycin (1 µM, measuring points 4-6), protonophore FCCP (1 µM, measuring points 7-9) and electron transport chain inhibitors antimycin A and rotenone (1 µM each, measuring points 10-12). (b) OCRs after 24 h of SM6 and SM8 treatment of human iPSC line NP0040 (mean ± SEM; n=7). (c) OCRs after 48 h of SM treatment of miPSC-CMs (mean ± SEM; n=7). (d) OCRs in miPSC-CMs after 48 h of SM treatment and 72 h of recovery after treatment (mean ± SEM; n=7).

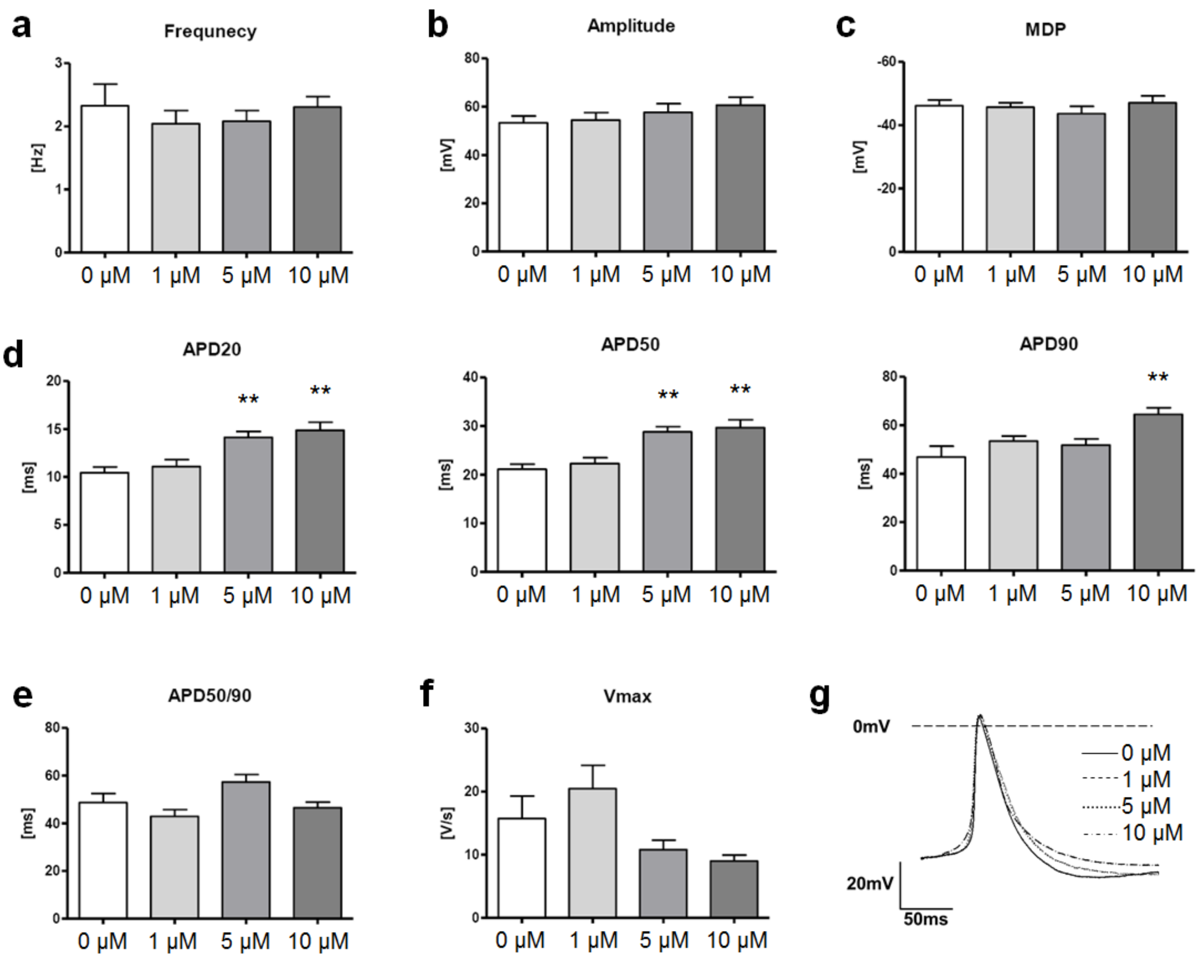

Figure S6. Action potential (AP) properties of αPIG-AT25 miPSC-derived day 16 cardiac clusters treated with SM6. Clusters were treated with concentrations of 0 µM (0.05% DMSO, n=27), 1 µM (n=38), 5 µM (n=27) and 10 µM (n=31) of SM6 for 48 h. Data are presented as mean ± SEM. (a) Beating frequency. (b) Amplitude. (c) Maximal diastolic potential (MDP). (d) Action potential durations at 20% (ADP20), 50% (ADP50) and 90% (ADP90) of depolarization. (e) Ratio of ADP50 and ADP90 (ADP50/90). (f) Maximal upstroke velocity (Vmax). (g) Overlay of APs treated with SM6 compared to untreated control.

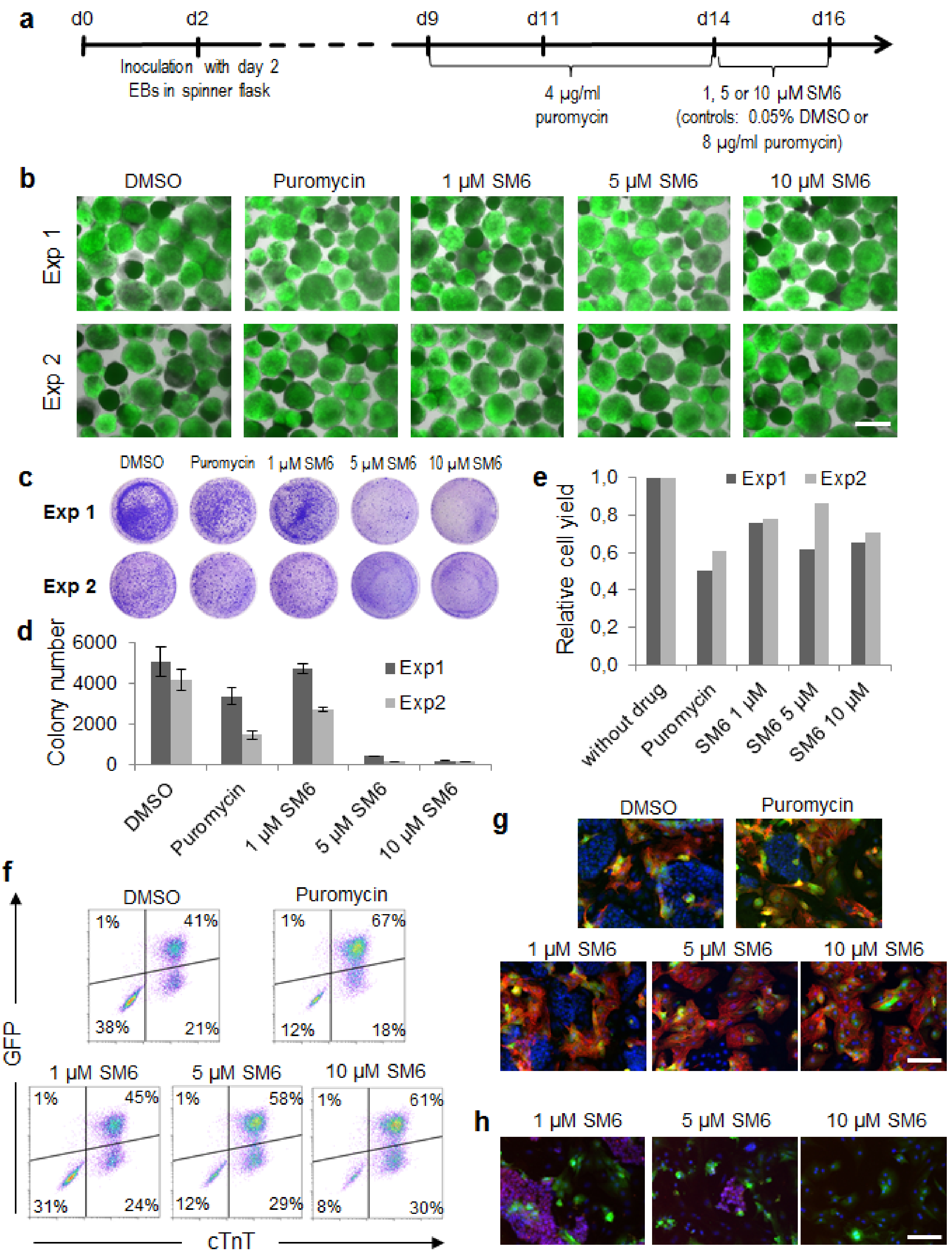

Figure S7. Selective elimination of PSCs in pre-purified αPIG-D3 mESC-derived cardiac clusters with SM6. (a) Time course of mESC cardiogenic differentiation and small molecule treatment. (b) Cardiac clusters from day 16 of differentiation (scale bar: 500 µm). (c) Cristal violet staining and (d) quantification of PSC-derived colonies after plating 2×10^5 cells derived from dissociated day 16 cardiac clusters (mean ± SD; n=3). (e) Relative cell yield in cardiac clusters from day 16 of differentiation compared to DMSO-treated control. (f) Flow

cytometric analysis of day 16 CMs immunostained for cardiac troponin T (cTnT). (g) ICC staining of α-actinin in CMs derived from dissociated cardiac clusters from day 16 of differentiation (blue: nuclei (Hoechst 33342), green: GFP, red: α-actinin; scale bar: 100 μm). (h) ICC staining of Oct4 in PSCs within the population of plated cells derived from SM6-treated cardiac clusters from day 16 of differentiation (blue: nuclei stained with Hoechst 33342, green: GFP, red: Oct4. scale bar: 100 μm).

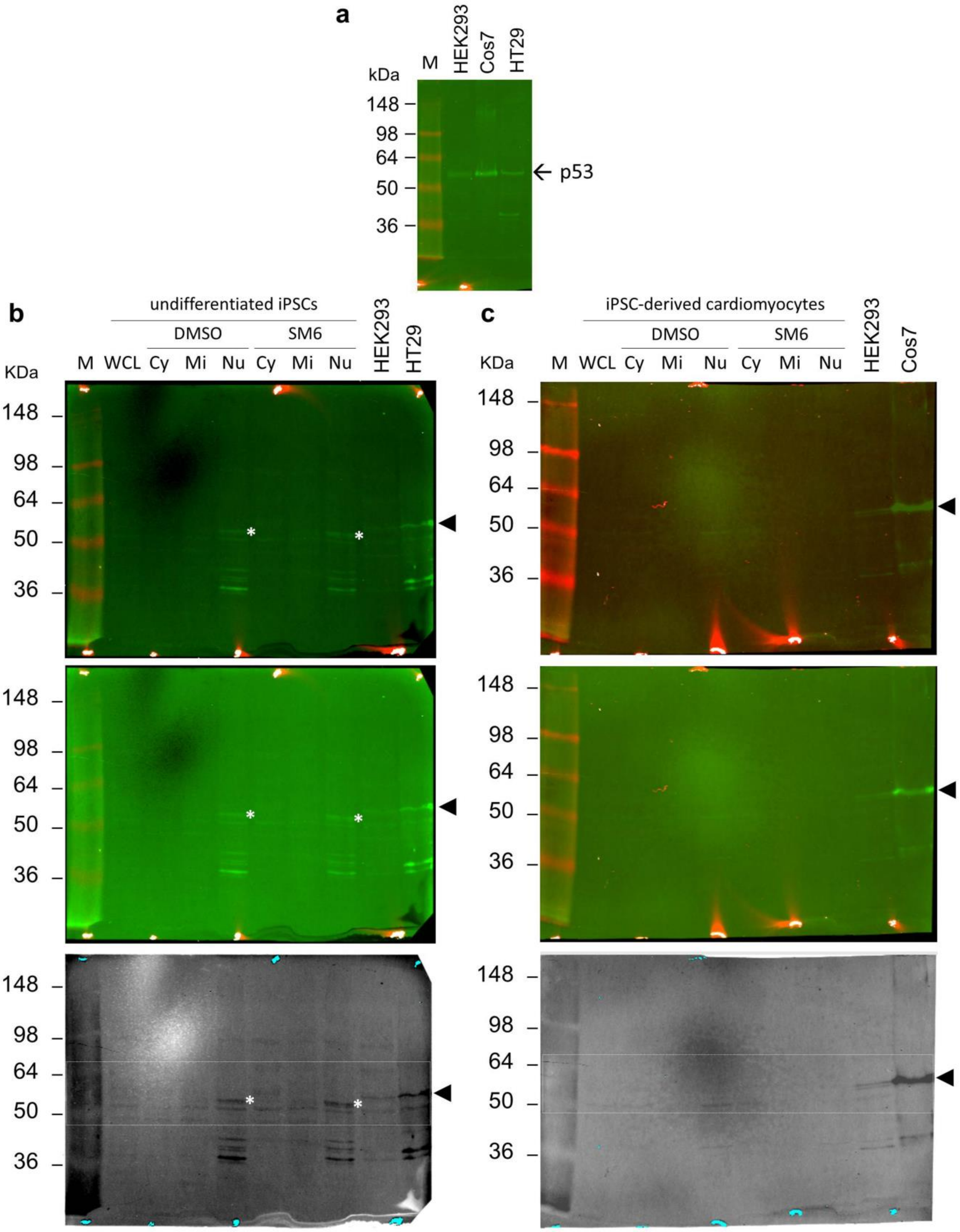

Figure S8. Western blot analysis of p53 expression in control cell lysates (a) and subcellular fractions of undifferentiated miPSCs (b) and miPSC-derived cardiomyocytes (c). miPSCs and miPSC-CMs were treated with 10 µM SM6 or vehicle (0.05% DMSO) for 8 h and then fractionated into cytoplasmic (Cy), mitochondrial (Mi) and nuclear (Nu) fractions. Protein samples were resolved by SDS-PAGE and transferred to PVDF membrane. Blots were probed with mouse monoclonal antibody against p53 (Cell Signaling Technology, Cat. No. 2524) followed by detection with DyLight 800 donkey anti-mouse IgG (Thermo Fisher). Near-infrared (NIR) fluorescence signals were detected on Odyssey CLx Gel Imaging

Scanner (LI-COR). (a) Full length immunoblot showing p53 (←) expression in whole cell lysates (WCL) of human HEK293, HT29 and COS9 cells which served as positive controls for p53 expression. (b, c) Full length immunoblots showing p53 expression in undifferentiated miPSCs (b) and miPSC-derived cardiomyocytes (c). p53 levels were determined in each subcellular fraction and in WCLs of miPSCs and miPSC-CMs. Since the molecular weight of human p53 is slightly higher than that of murine p53, the band representing this protein occupies slightly different position in murine (*) and human (◄) samples. The upper two blots show NIR fluorescent scans at two different exposures and the blot at the bottom of each panel represents the black & white rendition of the corresponding NIR fluorescence image. Blots shown in the Figure 4b in the main manuscript were cropped as indicated by white lines. Abbreviations: KDa – kilodalton; M – protein marker (SeeBlue Plus 2).

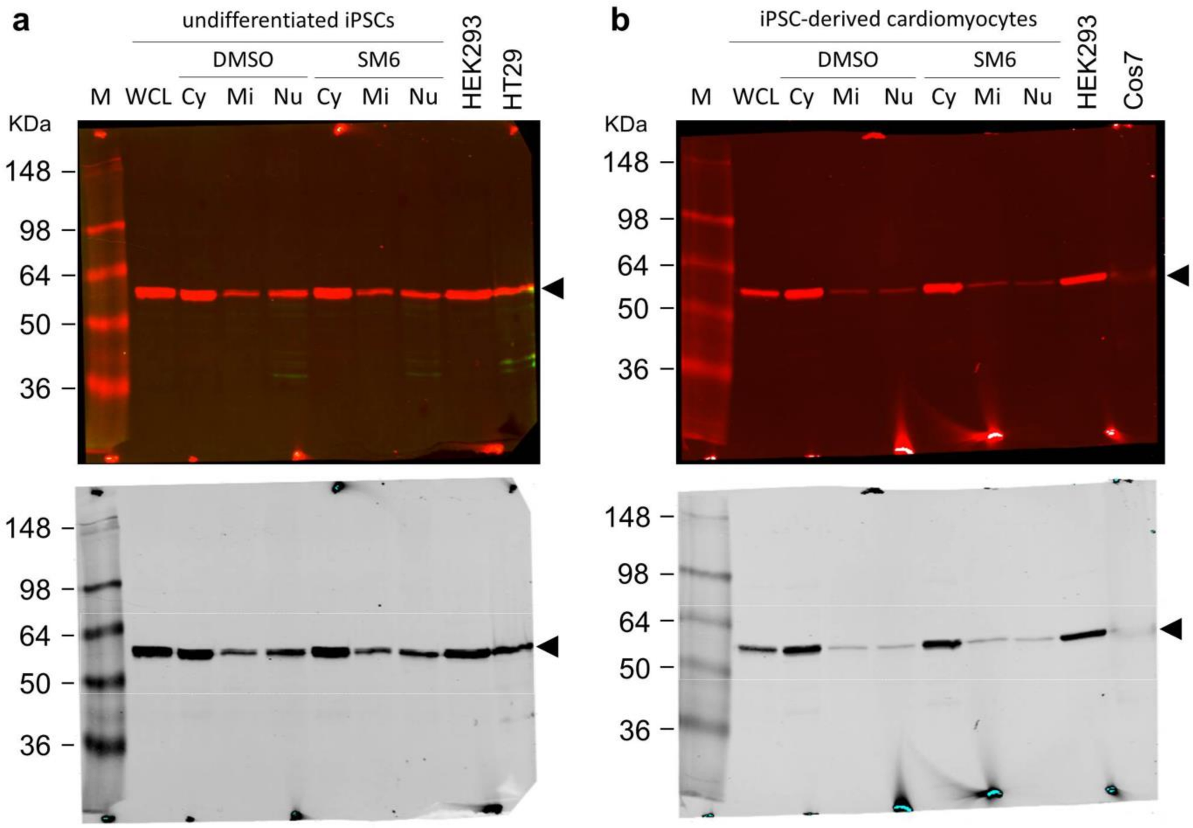

Figure S9. Western blot analysis of α-tubulin expression in whole cell lysates (WCL) and subcellular fractions of undifferentiated miPSCs (a) and miPSC-derived cardiomyocytes (b). miPSCs and miPSC-CMs were treated with 10 µM SM6 or vehicle (0.05% DMSO) for 8 h. Cells were then lysed to prepare WCLs or fractionated into cytoplasmic (Cy), mitochondrial (Mi) and nuclear (Nu) fractions. Protein samples were resolved by SDS-PAGE and transferred to PVDF membrane. Blots were probed with rabbit monoclonal antibody against α-tubulin (Cell Signaling Technology, Cat. No. 2125) followed by detection with IRDye 680RD donkey anti-rabbit IgG (Li-Cor). Near-infrared (NIR) fluorescence signals was detected on Odyssey CLx Gel Imaging Scanner (LI-COR). (a, b) Full length immunoblots showing α-tubulin (◀) expression in WCLs and subcellular fractions of undifferentiated miPSCs (a) and miPSC-derived cardiomyocytes (b). WCLs of human HEK293, HT29 and COS9 cells were used as controls. The upper blots in each panel show NIR fluorescence scans and the blots at the bottom represent the black & white rendition of the corresponding NIR fluorescence image. Blots shown in the Figure 4b in the main manuscript were cropped as indicated by white lines. *Abbreviations:* KDa – kilodalton; M – protein marker (SeeBlue Plus 2).

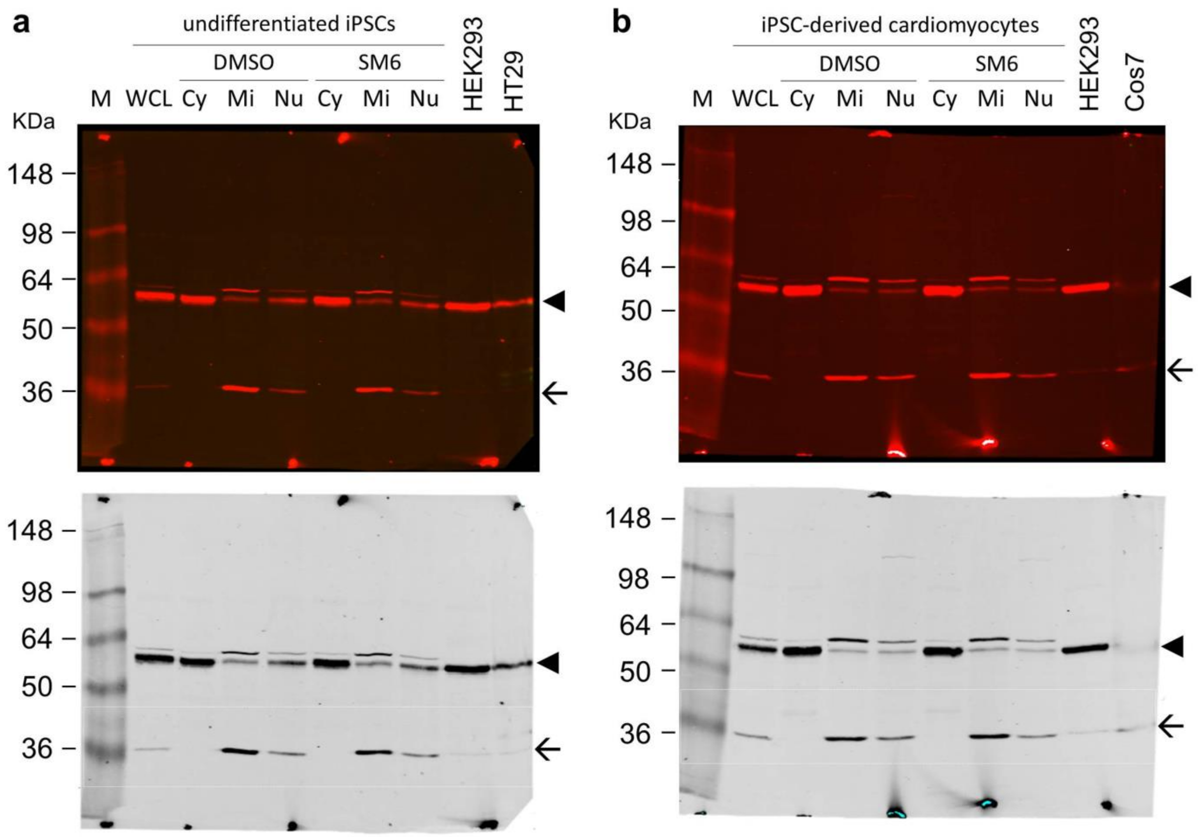

Figure S10. Western blot analysis of voltage-dependent anion channel (VDAC) expression in whole cell lysates (WCL) and subcellular fractions of undifferentiated miPSCs (a) and miPSC-derived cardiomyocytes (b). Protein samples and blots were prepared as described in the legend for Figure S9. Blots were probed with rabbit monoclonal antibody against the cytosolic marker α-tubulin (Cell Signaling Technology, Cat. No. 2125) and rabbit monoclonal antibody against the mitochondrial marker VDAC (Cell Signaling Technology, Cat. No. 4661) followed by detection with IRDye 680RD donkey anti-rabbit IgG (Li-Cor). Near-infrared (NIR) fluorescence signals was detected on Odyssey CLx Gel Imaging Scanner (LI-COR). (a, b) Full length immunoblots showing α-tubulin (◀) and VDAC (🡐) expression in WCLs and cytosolic (Cy), mitochondrial (Mi) and nuclear (Nu) fractions of undifferentiated miPSCs (a) and miPSC-derived cardiomyocytes (b). WCLs of human HEK293, HT29 and COS9 cells were used as controls. The upper blots in each panel show NIR fluorescence scans and the blots at the bottom represent the black & white rendition of the corresponding NIR fluorescence image. Blots shown in the Figure 4b in the main manuscript were cropped as indicated by white lines. *Abbreviations:* KDa – kilodalton; M – protein marker (SeeBlue Plus 2).

Supplementary Tables

Table S1: Characteristics of human iPSC lines used in this study.

hiPSC line (hPSC^{reg} name)*	Reprogramming method (somatic cell)	Donor age (sex)	Disease	Karyotype	Reference
NP0014-6 (UKKi007-A)	Retroviral (dermal fibroblasts)	45-49 (female)	CPVT1	Normal, 46, XX	Fatima, A. et al., 2011; Zhang X. H. et al., 2013
NP0040-8 (UKKi011-A)	Episomal (dermal fibroblasts)	35-39 (male)	Healthy	Normal, 46, XY	Hamad, S. et al., 2019; Zhang X. H. et al., 2019
NP0141-31B (UKKi032-C)	Sendai virus (PBMC)	40-44 (female)	Healthy	Normal, 46, XX	Hamad, S., et al., 2019

* hPSCreg – human PSC registry (https://hpscreg.eu).

Abbreviations: CPVT1 - Catecholaminergic polymorphic ventricular tachycardia, type 1; PBMC – peripheral blood mononuclear cells.

Table S2: Half maximal inhibitory concentration (IC_{50}*) of SMs for the indicated human iPSC lines after 48 h of treatment (mean ± SEE†; n=4).

Compound	NP0014-6 hiPSC	NP0040-8 hiPSC	NP0141-31B hiPSC
SM2	2.70±1.25 µM	5.45±0.86 µM	11.01±0.63 µM
SM6	1.56±0.71 µM	7.67±0.46 µM	**12.48±1.32** µM
SM8	**0.29±0.19 µM**	0.39±0.04 µM	0.44±0.04 µM

* The values marked in color indicate the lowest (red) and the highest (blue) IC_{50} values for human iPSCs.

† SEE - standard errors of the estimate. SEE values were calculated using standard errors of regression derived from logarithm-transformed regression analyses of dose response data.

Table S3: Half maximal inhibitory concentration (IC_{50}*) of SMs for one murine iPSC and two murine ESC lines after 48 h of treatment (mean ± SEE†; n=4-20).

Compound	αPIG-AT25 miPSC	αPIG-D3 mESC	R1 mESC
SM1	0.68±0.40 µM	0.43±0.15 µM	0.58±0.28 µM
SM2	1.03±1.09 µM	0.97±0.58 µM	**0.20±0.17 µM**
SM4	0.60±0.46 µM	0.63±0.25 µM	0.60±0.30 µM
SM5	0.97±0.87 µM	1.04±0.55 µM	0.59±0.33 µM
SM6	0.44±0.39 µM	0.53±0.23 µM	0.24±0.18 µM
SM8	0.78±0.44 µM	**1.10±0.41 µM**	0.26±0.14 µM

* The values marked in color indicate the lowest (red) and the highest (blue) IC_{50} values for murine PSCs.

† SEE - standard errors of the estimate. SEE values were calculated using standard errors of regression derived from logarithm-transformed regression analyses of dose response data.

Table S4. List of primary and matching secondary antibodies used for immunocytochemistry and immunoblotting.

Primary antibody	Provider	Cat. No.	Clone	Dilution of 1° Ab	Matching secondary antibody
Mouse α-actinin mAb, IgG1	Sigma-Aldrich	A7811	EA-53	1:800	Alexa Fluor 555 goat anti-mouse IgG1 (1:1000, Life Technologies, cat. no. A21127)
Rabbit Phospho-Histone H2A.X (Ser139) mAb, IgG	CST	9718	20E3	1:400	Alexa Fluor 555 goat anti-rabbit IgG (1:1000, Life Technologies, cat. no. A21428)
Rabbit cleaved caspase-3 mAb, IgG	CST	9664	5A1E	1:400	Alexa Fluor 555 goat anti-rabbit IgG (1:1000, Life Technologies, cat. no. A21428)
Mouse Oct4 mAb, IgG2b	Santa Cruz	sc-5279	C-10	1:400	Alexa Fluor 555 F(ab')2 goat anti-mouse IgG (1:1000, Life Technologies, cat. no. A21425)
Rabbit Nanog pAb, IgG	Santa Cruz	sc-33759	N/A	1:100	Alexa Fluor 488 goat anti-mouse IgG (1:1000, Life Technologies, cat. no. A11001)
Mouse SSEA-4 mAb, IgG3	Santa Cruz	sc-21704	813-70	1:200	Alexa Fluor 555 goat anti-rabbit IgG (1:1000, Life Technologies, cat. no. A21428)
Mouse TRA-1-81 mAb, IgMκ	Santa Cruz	sc-21706	N/A	1:200	Alexa Fluor 555 goat anti-mouse IgM (1:1000, Life Technologies, cat. no. A21426)
Rabbit α-Tubulin mAb	CST	2125	11H10	1:1000	Donkey anti-rabbit IgG, IRDye 680RD (1:10000, Li-Cor, cat. no. 926-68073)
Mouse p53 mAb	CST	2524	1C12	1:1000	Donkey anti-mouse IgG (H+L), DyLight 800 (1:10000, Thermo Fisher, cat. no. SA5-10172)
Rabbit VDAC mAb	CST	4661	D73D12	1:1000	Donkey anti-rabbit IgG, IRDye 680RD (1:10000, Li-Cor, cat. no. 926-68073)

* Abbreviations: mAb – monoclonal antibody, pAb – polyclonal antibody, CST - Cell Signaling Technology.

Supplementary References

Ben-David U., and Benvenisty N. Chemical ablation of tumor-initiating human pluripotent stem cells. *Nat Protoc* **9,** 729-740 (2014).

Ben-David U., Gan Q.F., Golan-Lev T., Arora P., Yanuka O., Oren Y.S., Leikin-Frenkel A., Graf M., Garippa R., Boehringer M., *et al.* Selective elimination of human pluripotent stem cells by an oleate synthesis inhibitor discovered in a high-throughput screen. *Cell Stem Cell* **12,** 167-179 (2013).

Berkessel A., Brandenburg M., Leitterstorf E., Frey J., Lex J., and Schäfer M. A Practical and Versatile Access to Dihydrosalen (Salalen) Ligands: Highly enantioselective titanium in situ catalysts for asymmetric epoxidation with aqueous hydrogen peroxide. *Adv Synth Catal* **349,** 2385-2391 (2007).

Butsch K., Günther T., Klein A., Stirnat K., Berkessel A., and Neudörfl, J. Redox chemistry of copper complexes with various salen type ligands. *Inorganica Chim Acta* **394,** 237-246 (2013).

Cabello N., Kizirian J.-C., Gille S., Alexakis A., Bernardinelli G., Pinchard L., and Caille J.-C. Simple 1,2-Diamine ligands for asymmetric addition of aryllithium reagents to imines. *Eur J Org Chem* **2005,** 4835-4842 (2005).

England J and Loughna S. Heavy and light roles: myosin in the morphogenesis of the heart. Cellular and molecular life sciences : *CMLS* **70**, 1221-1239 (2013).

Eröksüz S., Neudörfl J.M., and Berkessel, A. Kinetic resolution of 5-substituted oxazinones with bifunctional chiral base/squaramide organocatalysts. *Synlett* **28,** 1278-1281 (2017).

Fatima A., Xu G., Shao K., Papadopoulos S., Lehmann M., Arnaiz-Cot J. J., Rosa A. O., Nguemo F., Matzkies M., Dittmann S., Stone S. L., Linke M., Zechner U., Beyer V., Hennies H. C., Rosenkranz S., Klauke B., Parwani A. S., Haverkamp W., Pfitzer G., Farr M., Cleemann L., Morad M., Milting H., Hescheler J., and Saric T. In vitro modeling of ryanodine receptor 2 dysfunction using human induced pluripotent stem cells. *Cell Physiol Biochem* **28**, 579-592 (2011).

Gajewy J., Gawronski J., and Kwit M. Asymmetric hydrosilylation of ketones catalyzed by complexes formed from trans-diaminocyclohexane-based diamines and diethylzinc. *Monatsh Chem* **143,** 1045-1054 (2012).

Halbach M., Pillekamp F., Brockmeier K., Hescheler J., Müller-Ehmsen J., and Reppel M. Ventricular slices of adult mouse hearts - a new multicellular in vitro model for electrophysiological studies. *Cell Physiol Biochem* **18,** 1-8 (2006).

Hamad S., Derichsweiler D., Papadopoulos S., Nguemo F., Saric T., Sachinidis A., Brockmeier K., Hescheler J., Boukens B. J., and Pfannkuche K. Generation of human induced pluripotent stem cell-derived cardiomyocytes in 2D monolayer and scalable 3D suspension bioreactor cultures with reduced batch-to-batch variations. *Theranostics* **9**, 7222-7238 (2019).

Jiménez C.A. and Belmar J.B. Synthesis of highly hindered polyanionic chelating ligands. *Tetrahedron* **61,** 3933-3938 (2005).

Kolossov E., Bostani T., Roell W., Breitbach M., Pillekamp F., Nygren J.M., *et al.* Engraftment of engineered ES cell-derived cardiomyocytes but not BM cells restores contractile function to the infarcted myocardium. *J Exp Med* **203**, 2315-2327, (2006).

Kropp E.M., Oleson B.J., Broniowska K.A., Bhattacharya S., Chadwick A.C., Diers A.R., Hu Q., Sahoo D., Hogg N., Boheler K.R., *et al.* Inhibition of an NAD(+) salvage pathway provides efficient and selective toxicity to human pluripotent stem cells. *Stem Cells Transl Med* **4,** 483-493 (2015).

Lee M.O., Moon S.H., Jeong H.C., Yi J.Y., Lee T.H., Shim S.H., Rhee Y.H., Lee S.H., Oh S.J., Lee M.Y., *et al.* Inhibition of pluripotent stem cell-derived teratoma formation by small molecules. *Proc Natl Acad Sci U S A* **110,** E3281-3290 (2013).

Paton C.M. and Ntambi J.M. Biochemical and physiological function of stearoyl-CoA desaturase. *Am J Physiol Endocrinol Metab* **297,** E28-37 (2009).

Zhang X. H., Haviland S., Wei H., Saric T., Fatima A., Hescheler J., Cleemann L. and Morad M. Ca2+ signaling in human induced pluripotent stem cell-derived cardiomyocytes (iPS-CM) from normal and catecholaminergic polymorphic ventricular tachycardia (CPVT)-afflicted subjects. Cell Calcium 54, 57-70 (2013).

Zhang X. H., Saric T., Mehrjardi N. Z., Hamad S., and Morad M. Acid-sensitive ion channels are expressed in human induced pluripotent stem cell-derived cardiomyocytes. *Stem Cells Dev* **28**, 920-932 (2019).

Zhu Y., Malerich J.P., and Rawal V.H. Squaramide-catalyzed enantioselective Michael addition of diphenyl phosphite to nitroalkenes. *Angew Chem* **49,** 153-156 (2010).
